# Assessment of *Cryptosporidium* spp. Sub-Families and *Giardia duodenalis* Assemblages A and B in Ghanaian HIV Patients, Including Socio-Economic, Clinical, and Immunological Associations

**DOI:** 10.3390/idr17050129

**Published:** 2025-10-15

**Authors:** Lynn Glyschewski, Hagen Frickmann, Fred Stephen Sarfo, Betty Roberta Norman, Albert Dompreh, Emmanuel Acheamfour-Akowuah, Martin Kofi Agyei, Shadrack Osei Asibey, Richard Boateng, Edmund Osei Kuffour, Veronica Di Cristanziano, Sven Poppert, Felix Weinreich, Albert Eisenbarth, Tafese Beyene Tufa, Torsten Feldt, Kirsten Alexandra Eberhardt

**Affiliations:** 1 Department of Microbiology and Hospital Hygiene, Bundeswehr Hospital Hamburg, 20359 Hamburg, Germany; lynn.glyschewski@stud.uke.uni-hamburg.de (L.G.); albert.eisenbarth@bnitm.de (A.E.); 2 Institute for Medical Microbiology, Virology and Hygiene, University Medicine Rostock, 18057 Rostock, Germany; 3 Department of Medicine, Komfo Anokye Teaching Hospital, Kumasi 00233, Ghana; stephensarfo78@gmail.com (F.S.S.); branorman@yahoo.com (B.R.N.); a.acheamfour@yahoo.com (E.A.-A.); martinagyei@yahoo.co.uk (M.K.A.); shakosbey19@gmail.com (S.O.A.); 4 Kwame Nkrumah University of Science and Technology, Kumasi 00233, Ghana; 5 Department of Clinical Microbiology, Komfo Anokye Teaching Hospital, Kumasi 00233, Ghana; adompreh@gmail.com (A.D.); richardboateng166@gmail.com (R.B.); 6 Laboratory of Retrovirology, The Rockefeller University, New York, NY 10065, USA; eosei@rockefeller.edu; 7 Institute of Virology, Faculty of Medicine and University Hospital Cologne, University of Cologne, 50937 Cologne, Germany; veronica.di-cristanziano@uk-koeln.de; 8 Independent Researcher, 13353 Berlin, Germany; sven@poppert.eu; 9 Department of Laboratory Medicine, Bundeswehr Central Hospital Koblenz, 56072 Koblenz, Germany; felixweinreich@bundeswehr.org; 10 Hirsch Institute of Tropical Medicine (HITM), Heinrich-Heine University, Asella P.O. Box 04, Ethiopia; tafeseb.tufa@yahoo.com; 11 Department of Gastroenterology, Hepatology and Infectious Diseases, Medical Faculty and University Hospital Düsseldorf, Heinrich Heine University Düsseldorf, 40225 Düsseldorf, Germany; torsten.feldt@med.uni-duesseldorf.de (T.F.);; 12 Department of Tropical Medicine, Bernhard Nocht Institute for Tropical Medicine & I. Department of Medicine, University Medical Center Hamburg-Eppendorf, 20359 Hamburg, Germany

**Keywords:** enteric infection, giardiasis, assemblage, immunosuppression, Africa, cryptosporidiasis, sequence typing, epidemiology

## Abstract

**Background**: *Cryptosporidium* spp. cause opportunistic infections in immunosuppressed individuals, such as people living with HIV (PLWH). However, the association between giardiasis and HIV infection remains uncertain. This study assessed co-infections in Ghanaian PLWH and HIV-negative individuals, analyzing socio-economic, clinical, and immunological implications, including the *Giardia duodenalis* assemblage and *Cryptosporidium* spp. sub-family levels. **Methods:** Stool samples from Ghanaian PLWH were tested using several real-time PCR assays targeting *G. duodenalis* at the species level and assemblages A and B to optimize diagnostic accuracy. GD60 gene-based Sanger sequencing was used for *Cryptosporidium* spp. subtyping. Results were correlated with anonymized patient data to evaluate interactions with HIV infection. **Results:** In PLWH, *C. hominis* Ib, *C. hominis* Ie, and *C. parvum* IIc were detected at similar frequencies, followed by *C. hominis* Ia, *C. hominis* Id, and *C. parvum* IIe in decreasing order. Only *C. parvum* IIc was repeatedly observed in individuals with CD4+ T cell counts above 200/µL, while other sub-families occurred preferentially in those with lower counts. *C. hominis* Ia and Ib were associated with PLWH not receiving antiretroviral therapy; *C. hominis* Ia was linked to recently diagnosed HIV infections. No relevant associations between *G. duodenalis* assemblages and HIV infection were found. **Conclusions:** Sub-families Ia and Ib of *C. hominis* preferentially occur in individuals with severe immunosuppression, while *C. parvum* IIc is also detectable in individuals with better immune function. The prevalence of giardiasis in Ghana appears to be influenced by factors other than HIV-induced immunosuppression.

## 1. Introduction

*Cryptosporidium* spp. are protozoan parasites that cause opportunistic enteric infections in humans and various non-human vertebrates, depending on host susceptibility [1]. Besides immunosuppressed individuals, such as people living with HIV (PLWH), children under two years of age are particularly at risk of infection in endemic areas, often due to poor hygiene conditions [2]. Infection typically occurs through contaminated water sources [3], and the parasite’s oocysts are highly resistant to environmental inactivation [4]. Recent evidence also suggests associations with sexually active populations and potential sexual transmission routes, especially among men who have sex with men (MSM) [5]. Therapeutic options remain limited, particularly when the patient’s immune status cannot be improved. Although drugs like paromomycin and nitazoxanide have been used, their efficacy remains unconvincing [4].

In contrast to *Cryptosporidium* spp., the relationship between HIV infection and enteric infection or colonization with the protozoan parasite *Giardia duodenalis* remains controversial and uncertain [6]. Studies from Sub-Saharan Africa report a likely association between HIV and increased *G. duodenalis* co-infection rates in children, with a meta-analysis suggesting a pooled prevalence of 25.7% in this subgroup [7]. Similar trends have been observed in Asia among PLWH [8]. Historically, HIV enteropathy and AIDS-related diarrhea have been linked to *G. duodenalis* among other pathogens [9,10]. However, a global review on giardiasis in HIV patients found only a slightly to moderately increased odds ratio (OR) of 1.7 for giardiasis in co-infected individuals compared to HIV-negative persons, with a pooled prevalence close to 5% [11]. Importantly, factors such as diarrhea showed a stronger association, increasing the likelihood of *G. duodenalis* carriage by 3.8 times. The review also indicated a negligible influence of immune competence on giardiasis prevalence, with little difference in the OR between HIV patients on anti-retroviral therapy (ART) compared to HIV patients not receiving this treatment [11]. Potential explanations for a link between giardiasis and HIV include sexual routes shared by both infections [12]. However, this does not explain the high co-infection rates in young African children unlikely to engage in sexual activity [7]. A small US study suggested that HIV patients co-infected with *G. duodenalis* exhibited higher leukocyte counts and a trend toward lower CD4+ T-lymphocytes. In 24.1% of co-infected individuals, initial metronidazole treatment failed, with increased blood hemoglobin levels uniquely associated with treatment failure [13]. Generally, low CD4+ T-lymphocyte counts are risk factors for severe, disseminated, and atypical protozoan infections, including giardiasis [14]. Impaired antibody responses to *G. duodenalis* are also considered a risk for poor immunological control [15]. Nevertheless, *G. duodenalis* infection in HIV patients is usually acute and painful but rarely chronic due to effective antimicrobial treatment [15]. Despite ongoing debate on HIV’s role in giardiasis, there is limited information on *G. duodenalis* assemblages below the species level [15,16,17,18], especially regarding assemblages A and B, in particular, which are linked to zoonotic and human infections but lack data on HIV associations [16,19].

The quality of prevalence data for protozoan infections like cryptosporidiosis and giardiasis also depends heavily on diagnostic accuracy. Various diagnostic methods exist, including light and fluorescence microscopy, as well as molecular tools [3]. *Cryptosporidium* spp. show high genetic diversity, with dozens of species and over 100 genotypes described [20]. Sanger sequencing of the GD60 gene is a traditional typing method developed over 20 years ago [21,22] and remains in use in recent Ghanaian studies [23]. Stool microscopy is less sensitive than real-time PCR for detecting *G. duodenalis* [24], but PCR-based assays also face challenges with suboptimal sensitivity and specificity, including those targeting specific *G. duodenalis* assemblages [25]. Thus, these limitations should be considered when planning studies on HIV and parasitic co-infections.

In Ghana, PLWH and children are among the groups most susceptible to cryptosporidiosis, with prevalence estimates ranging from 5% to 10% in cross-sectional studies [23,26]. GD60 gene typing in Ghanian children has identified sub-families IIc, Ib, and Ia as the most frequent for *C. hominis* (Ib, Ia) and *C. parvum* (IIc) [23]. The present study aims to explore associations between giardiasis overall, *G. duodenalis* assemblages A and B in particular, and sequence-confirmed *Cryptosporidium* spp. sub-families with socio-economic, clinical, and immunological factors in Ghanaian PWLH. *Cryptosporidium* spp. are included as a well-established parameter linked to HIV infection, while such associations are controversial for giardiasis. This exploratory approach seeks to clarify both established and uncertain interactions. To address issues of diagnostic accuracy, multiple *Giardia*-specific real-time PCR assays were employed alongside GD60 gene Sanger sequencing of *Cryptosporidium*-positive samples, as detailed in the Methods. Ultimately, this study aims to contribute valuable epidemiological insights into cryptosporidiosis and giardiasis within Ghanaian PLWH as an example of a lower-income setting.

## 2. Materials and Methods

### 2.1. Study Design and Sample Materials

PLWH attending the HIV outpatient department at the Komfo Anokye Teaching Hospital (Kumasi, Ghana) were invited to participate in this cross-sectional study, which focused on associations between gastrointestinal and other pathogens and socio-demographic, clinical, and immunological parameters. For comparison, HIV-negative adults from the same region were also included [27,28]. Equal numbers of PLWH receiving and not receiving antiretroviral therapy ensured the assessment of possible therapy effects. The entire study population, comprising both HIV-positive and HIV-negative individuals, was investigated over a 12-month period. Demographic, socio-economic, immunological, and clinical data were collected using standardized questionnaires administered by trained investigators.

### 2.2. Laboratory Diagnostics

Venous blood samples were used for CD4+ T lymphocytes counts, performed with a FACSCalibur flow cytometer (Becton Dickinson, Mountain View, CA, USA) in Ghana. HIV-1 viral loads were measured with a Real-Time HIV-1 PCR system (Abbott Diagnostics, Wiesbaden, Germany).

Peripheral blood mononuclear cells (PBMCs) were isolated from heparinized venous blood by Ficoll/Hypaque-based density gradient centrifugation (Biocoll Separating Solution, Biochrom AG, Berlin, Germany), washed with phosphate-buffered saline, and resuspended in Roswell Park Memorial Institute 1640 medium (Gibco Invitrogen, Carlsbad, CA, USA) supplemented with heat-inactivated fetal calf serum (Biochrom AG, Berlin, Germany). Cryopreserved PBMCs were shipped in liquid nitrogen to Germany for cell surface marker staining, as previously reported [28]. Cytometric results were obtained using an LSRII flow cytometer (BD Biosciences, Heidelberg, Germany) and analyzed with FlowJo (version 9.6.2, Tree Star, San Carlos, CA, USA).

Native stool aliquots were stored at −80 °C until DNA extraction, which was performed using the QIAamp stool DNA mini kit (Qiagen, Hilden, Germany) according to the manufacturer’s instructions. In a previous investigation, stool samples had been assessed with three different real-time PCR assays for *Cryptosporidium* spp., which showed sensitivities of 88.8–100% and specificities of 96.9–99.6% [29]. Samples with at least one positive signal in this prior assessment were included and reanalyzed with a nested PCR on a LightCycler Pro device (Roche, Basel, Switzerland), as detailed in Appendix A, Table A1 and Table A2. Each run included a plasmid-based positive control (sequence inserts in a pEX A128 vector backbone (Eurofins Genomics, Luxembourg)) and a PCR-grade water-based negative control for quality control (Appendix A, Table A1). Amplicons were visualized using a Lonza FlashGel system (Lonza Group, Basel, Switzerland). Gel-positive samples were submitted for nucleic acid extraction and Sanger sequencing to Microsynth Seqlab GmbH (Göttingen, Germany). The same primers used for the secondary PCR reaction (see Appendix A, Table A1, for details) were applied for sequencing. Forward and reverse strand sequences were manually aligned, and quality control was performed using the Finch TV software (Version 1.4, Geospiza Inc., 2004–2012, Seattle, WA, USA). Because sequencing was solely diagnostically performed and not associated with sample-associated characteristics, no sequence files were deposited at international databases. Instead, aligned sequence raw reads are provided in the Appendix A section of this article (please also see the Results for details). *Cryptosporidium* spp. sub-families were identified using the NCBI GenBank database [30].

For molecular diagnosis of giardiasis, laboratory-developed real-time PCR assays from the literature [25,31,32,33,34,35] were applied to detect *G. duodenalis* generally, as well as assemblages A and B. These targeted the following: a 75-base pair sequence of the beta giardin (*bg*) gene (referred to as *G. duodenalis* PCR 1), a 63-base pair sequence of the 18S rRNA gene (referred to as *G. duodenalsis* PCR 2), a 98-base pair sequence of the glutamate dehydrogenase (*gdh*) gene (referred to as *G. duodenalis* PCR 3) of *G. duodenalis*, a 75-base pair sequence of the *bg* gene amplified by assays with (referred to as *G. duodenalis* assemblage A PCR 1) and without (referred to as *G. duodenalis* assemblage A PCR 2) the use of hybridization probes containing locked nucleic acids, and a 76-base pair sequence of the triosephosphate isomerase (*tpi*) gene of of *G. duodenalis* assemblage A, as well as a 75-base pair sequence of the *bg* gene amplified by assays with (referred to as *G. duodenalis* assemblage B PCR 1) and without (referred to as *G. duodenalis* assemblage B PCR 2) the use of hybridization probes containing locked nucleic acids, and a 82-base pair sequence of the triosephosphate isomerase (*tpi*) gene of *G. duodenalis* assemblage B. Assay performance estimates included sensitivities of 17.5–100%, specificities of 84–100%, and limits of detection ranging from <10 to 83 copies per µL eluate, with kappa between 0.155 and 0.908 [25]. Each PCR run included negative (PCR-grade water) and positive (plasmid target sequence in a pEX-A128 vector) controls. Appendix A, Table A3, details oligonucleotide sequences and assay limits of detection. Real-time PCR assays were conducted on Corbett Q cyclers (Qiagen, Hilden, Germany), using master mix compositions and cycling conditions provided in Appendix A, Table A4. To control for sample inhibition, a Phocid herpes virus (PhHV) DNA-specific real-time PCR was used [36].

### 2.3. Case Definitions and Exclusion and Inclusion Criteria

For *Cryptosporidium* spp. sub-family identification, only samples with sequences of sufficient quality for sub-family-level assignment were considered positive. Given limitations in giardiasis diagnostic accuracy [25], samples were only considered truly positive if at least two out of three *G. duodenalis*-specific assays yielded positive results; single positive signals were excluded as likely false positives. Only confirmed *G. duodenalis*-positive samples were further tested for assemblages A and B using additional real-time PCR assays. Again, positive results in at least 2 out of 3 assemblage-specific assays defined true positives; a lone positive was considered a likely false positive. Samples positive for *G. duodenalis* but unassignable to assemblage A or B were classified as non-A-non-B assemblages. Duplicate samples from the same patient were excluded unless a new positive result was detected.

Cycle threshold (Ct) values for *G. duodenalis*-specific real-time PCR were categorized into three semi-quantitative ranges: “High target DNA amount” (Ct < 25), “intermediate target DNA amount” (Ct 25–35), and “low target DNA amount” (Ct > 35). Assignment to these categories was based on the lowest Ct value recorded for each sample. Semi-quantitative assessment was limited to giardiasis, as nested PCR for *Cryptosporidium* typing did not yield Ct values.

### 2.4. Statistics

Continuous variables were summarized as median and interquartile range (IQR) and compared using the Kruskal–Wallis test. Categorical variables were analyzed with the generalized Fisher’s exact test. Pairwise post hoc tests with false discovery rate (FDR) correction were conducted for variables with significant results. Multiple linear regression analysis was performed using the R package (Version 4.4.3, R Foundation for Statistical Computing, Vienna, Austria) “forestmodel”. Associations between ordinal and continuous variables were assessed with Kendall’s rank correlation tau. Two-sided *p*-values were provided, with statistical significance set at α = 5%. All statistical analyses were conducted using R (version 4.4.3, R Foundation for Statistical Computing, Vienna, Austria). Notably, no sample size calculations were performed for this hypothesis-generating, exploratory analysis, which did not include any predefined effect size assumptions.

### 2.5. Ethics

The study was conducted in accordance with the Declaration of Helsinki and its amendments. Sample collection and analysis followed protocols approved by the Committee on Human Research of the Kwame Nkrumah University of Science and Technology in Kumasi, Ghana, CHRPE/AP/12/11 (approved on 8 September 2011), and the ethics committee of the Medical Council in Hamburg, Germany, PV3771 (approved 13 May 2011). Written informed consent was obtained from all participants.

## 3. Results

### 3.1. Application of the Exclusion and Inclusion Criteria to the Samples Tested

From 114 samples that had shown at least one positive *Cryptosporidium* spp.-specific real-time PCR signal in the previous assessment [29], amplification of a *Cryptosporidium* spp.-specific sequence allowing for discrimination at the sub-family level was achieved in 47 samples (Appendix A Table A5), which corresponded to 41 patients. Specifically, *C. hominis* sequences were assigned to 30 patient clusters: Ia (*n* = 4), Ib (*n* = 15), Id (*n* = 3), and Ie (*n* = 8). Among the remaining 11 patients, sequences were assigned to *C. parvum* clusters IIc (*n* = 8) and IIe (*n* = 3). Detailed sequence results and assignments are presented in Appendix A, Table A5.

Of those tested for molecular evidence of giardiasis, 26 samples were classified as true positives for *G. duodenalis* DNA, as they yielded positive results in at least two out of three *G. duodenalis*-specific real-time PCR assays. Signals regarded as likely false positives included 33 from *G. duodenalis* PCR 1, 64 from *G. duodenalis* PCR 2, and none from *G. duodenalis* PCR 3. Among the 26 true-positive samples for *G. duodenalis*, assemblage-specific PCR assessment identified 6 as assemblage A, 12 as assemblage B, and 8 as non-A-non-B assemblages. Assemblage-specific signals excluded as likely false positives comprised two for assemblage B PCR 2 and one each for assemblage A PCR 2 and assemblage B PCR 3; no such likely false positives were recorded for the remaining assays.

Table 1 presents a visualization of these findings. Notably, co-occurrence of *G. duodenalis* and *Cryptosporidium* spp. in the same sample was exceptionally rare. Specifically, *G. duodenalis* assemblage B and *C. hominis* Ib were detected together in one sample, and *G. duodenalis* assemblage B and *C. parvum* IIc in another. No clear evidence of clustering was observed.

### 3.2. Prevalence of DNA of the Various Cryptosporidium spp. Sub-Families as Well as G. duodenalis and Assemblages A and B Within the Stool Samples of the Study Population

**Figure 1:**
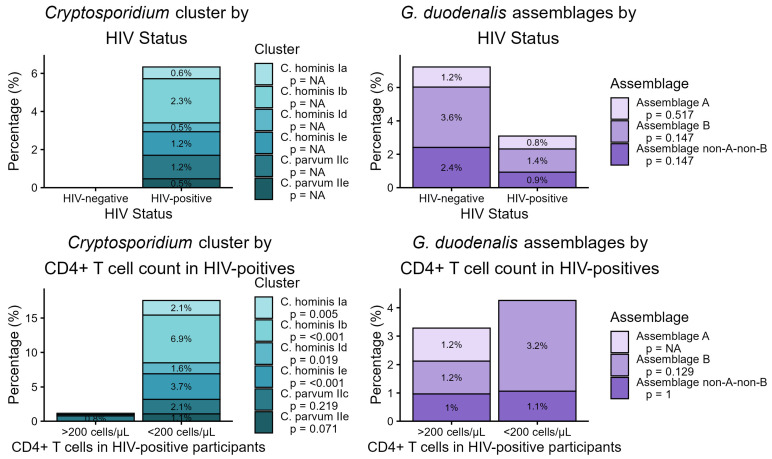
Prevalence of *Cryptosporidium* spp. sub-families, as well as *G. duodenalis* and assemblages A and B, according to the HIV status and stratified by CD4+ T-lymphocyte count. The letter p describes the significance level.

Among 730 stool samples analyzed, *G. duodenalis* DNA was detected in 26 cases (3.6%). Detection rates differed by HIV status: 6 out of 83 HIV-negative individuals (7.2%) tested positive, compared to 20 out of 647 HIV-positive individuals (3.1%, *p* = 0.105; Figure 1). Assemblage A was identified in six samples (0.8%), comprising one from an HIV-negative subject (1.2%) and five from HIV-positive subjects (0.8%; *p* = 0.517). Assemblage B was detected in twelve samples (1.6%), including three HIV-negative (3.6%) and nine HIV-positive individuals (1.4%, *p* = 0.147). Non-A-non-B assemblages accounted for eight cases (1.1%), with two detected among HIV-negative (2.4%) and six among HIV-positive participants (0.9%, *p* = 0.147).

*Cryptosporidium* spp. DNA was found exclusively in HIV-positive individuals, with 41 out of 647 participants (6.3%) testing positive. Analyzing sub-family prevalence by CD4+ T cell count showed significantly higher detection rates in those with counts below 200/µL. Specifically, *C. hominis* Ia was detected in 4 out of 188 (2.1%) with low CD4+ T cell counts and in none of the 518 with higher counts (*p* = 0.005); *C. hominis* Ib was observed in 13 out of 188 (6.9%) with low CD4+ counts versus 1 out of 518 (0.2%; *p* < 0.001); *C. hominis* Id was found in 3 out of 188 (1.6%) compared to none in those with higher counts (0.0%; *p* = 0.019). *C. hominis* Ie was present in 7 out of 188 (3.7%) versus 1 out of 518 (0.2%; *p* < 0.001). For *C. parvum* IIc, 4 out of 188 (2.1%) samples from individuals with low CD4+ T cell counts and 4 out of 518 (0.8%) samples from those with higher counts were positive (*p* = 0.219). A non-significant trend was noted for *C. parvum* IIe, with 2 out of 188 (1.1%) positive in the low CD4+ group and none found among participants with higher counts (*p* = 0.071).

### 3.3. Comparison of Demographic, Socio-Economic, and Clinical Characteristics of the HIV-Positive Cohort According to the Presence or Absence of DNA of the Various Cryptosporidium spp. Sub-Families, as Well as G. duodenalis and Assemblages A and B, in Stool Samples

Demographic, socio-economic, medical-treatment-related, and clinical characteristics of HIV-positive participants were evaluated according to the detection of *Cryptosporidium* spp. sub-families (Table 2). The median age was similar across groups, with no significant differences observed (*p* = 0.889). Female participants comprised 73.5% of those without detectable *Cryptosporidium* spp. DNA and varied among sub-families, though without statistically significant differences (*p* = 0.121). Access to tap water, household electricity, and refrigerator ownership showed no significant variation between groups (*p* = 0.341, 0.325, and 0.524, respectively). Intake of combination antiretroviral therapy (cART) differed significantly across sub-families (*p* = 0.001), notably with none of the participants in the Ia and Ib sub-groups receiving cART. Use of trimethoprim/sulfamethoxazole (TMP/SMX) prophylaxis showed a trend toward significance (*p* = 0.067), with higher proportions in certain sub-families. Body mass index did not differ significantly (*p* = 0.527). Duration since HIV diagnosis varied significantly (*p* = 0.005), with the shortest duration observed in the Ia sub-family group. Self-reported clinical symptoms within the previous six months revealed notable findings: cough was more frequently reported in some sub-families (*p* = 0.006), while diarrhea (*p* = 0.007), fever (*p* = 0.005), skin rash (*p* = 0.001), and weight loss (*p* = 0.001) also showed variation. Nonetheless, pairwise comparisons with FDR correction did not indicate statistically significant differences.

The demographic, socio-economic, medical treatment-related, and clinical characteristics of HIV-positive participants were also analyzed according to the detection of *G. duodenalis* assemblages (Table 3). The median age across groups showed no statistically significant difference, ranging from 34 (assemblage B) to 46 years (assemblage non-A-non-B) (*p* = 0.153). Females constituted approximately 73% of the group without detectable *G. duodenalis* DNA and ranged from 50% to 100% across the assemblage groups, without significant variation (*p* = 0.124). Access to tap water differed significantly between groups (*p* = 0.025); however, FDR-corrected pairwise comparisons did not reveal a significant pairwise difference. No significant differences were observed for household electricity access or refrigerator ownership (*p* = 1.000 and *p* = 0.324, respectively). The proportion of participants receiving cART and TMP/SMX prophylaxis did not differ significantly across assemblage groups (*p* = 0.756 and *p* = 0.241, respectively). Body mass index and time since HIV diagnosis also showed no significant differences (*p* = 0.678 and *p* = 0.497, respectively). Self-reported clinical symptoms during the preceding six months, including cough, diarrhea, fever, weight loss, and skin rash, were similar among groups. Although skin rash showed a significant difference in the overall comparison (*p* = 0.012), FDR-adjusted pairwise comparisons did not detect significant individual differences.

### 3.4. Comparison of Virological and Immunological Characteristics of HIV-Positive Participants Depending on the Abundance or Absence of DNA of the Various Cryptosporidium spp. Sub-Families, as Well as G. duodenalis and Assemblages A and B, in Their Stool Samples

Analysis of virological and immunological parameters revealed significant differences among HIV-positive participants, as stratified by the detection of *Cryptosporidium* spp. sub-families (Table 4). Median viral loads (log_10 copies/mL) were markedly higher in all *Cryptosporidium* spp.-positive groups compared to participants without detectable *Cryptosporidium* spp. DNA, ranging from 5.2 to 6.6 versus 4.1, respectively (*p* < 0.001). Pairwise comparisons adjusted for false discovery rate indicated statistically significant differences between the control group and sub-families Ia, Ib, Id, Ie, and IIc. Similarly, CD4+ T cell counts were significantly lower in all *Cryptosporidium* spp.-positive sub-groups, with medians ranging between 24 and 155 cells/µL compared to 359 cells/µL in participants without proof of *Cryptosporidium* spp. DNA (*p* < 0.001). Significant pairwise differences were observed for all sub-families except IIe. CD8+ T cell counts did not differ significantly among groups (*p* = 0.307). The CD4+/CD8+ T cell ratio showed pronounced reductions in *Cryptosporidium* spp.-infected participants, with median ratios between 0.02 and 0.12 versus 0.39 in the control group (*p* < 0.001). Significant pairwise contrasts were noted for all sub-families (Ia, Ib, Id, Ie, IIc, and IIe).

The distribution of virological and immunological parameters stratified by *G. duodenalis* assemblages in HIV-positive participants did not show statistically significant differences (Table 5). Median viral loads (log_10 copies/mL) were comparable across groups, ranging from 1.6 in the assemblage A group to 4.3 in participants without detectable *G. duodenalis* DNA (*p* = 0.556). CD4+ T-lymphocyte counts also did not differ significantly, with medians of 345 cells/µL for assemblage A, 116 for assemblage B, 265 for non-A-non-B assemblages, and 348 for participants without detectable *G. duodenalis* DNA (*p* = 0.153). Similarly, CD8+ T cell counts were consistent across groups (*p* = 0.915). The CD4+/CD8+ T cell ratio showed a non-significant trend toward variation (*p* = 0.067), with the highest median ratio observed in the assemblage A group (0.7) and the lowest in the group positive for assemblage B (0.1).

### 3.5. Factors Associated with Co-Infection with the Various Cryptosporidium spp. Sub-Families as Well as G. duodenalis and Assemblages A and B in the HIV-Positive Cohort

**Figure 2:**
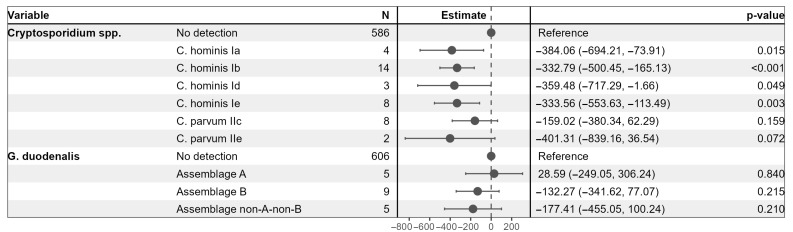
Multivariable linear regression model to examine the association of *Cryptosporidium* spp. sub-families and *G. duodenalis* assemblages with CD4+ T cell counts among HIV-positive participants.

A multivariable linear regression model was employed to examine associations between *Cryptosporidium* spp. sub-families and *G. duodenalis* assemblages and CD4+ T cell counts among HIV-positive participants (Figure 2). Using individuals without detectable *Cryptosporidium* spp. or *G. duodenalis* as reference groups, the model demonstrated that infection with several *Cryptosporidium* spp. sub-families was significantly associated with lower CD4+ T cell counts. Specifically, the presence of *C. hominis* sub-families Ia, Ib, Id, and Ie corresponded to reductions in CD4+ T cell counts ranging from approximately 333 to 384 cells/µL compared to the reference group (all *p* < 0.05). *C. parvum* sub-families IIc and IIe were associated with non-significant reductions. None of the *G. duodenalis* assemblages showed a significant association with CD4+ T cell counts in this model.

### 3.6. Correlations of Cycle Threshold (Ct) Values for Specific Sequences of the Various Cryptosporidium spp. Sub-Families as Well as G. duodenalis and Assemblages A and B with CD4+ T Cell Count, CD4+/CD8+ T Cell Ratio, and HIV Viral Load

Correlation analysis between Ct values from real-time PCR targeting *G. duodenalis* and its assemblages and immunological and virological parameters showed no statistically significant associations in the overall cohort (Table 6). Kendall’s tau coefficients indicated weak positive correlations between Ct values and CD4+ T cell counts (tau = 0.27 for overall *G. duodenalis* Ct values) and the CD4+/CD8+ T cell ratio (tau = 0.32), although these did not reach significance (*p* > 0.10). Viral load exhibited a weak negative correlation with Ct values (tau = −0.25), which was also non-significant (*p* = 0.227). Subgroup analyses of assemblages A and B demonstrated similar non-significant correlations across all immunological and virological parameters.

## 4. Discussion

The study aimed to provide epidemiological data on *Cryptosporidium* spp. sub-families and *Giardia duodenalis* assemblage in Ghanaian PLWH. Several key findings emerged.

Focusing on *Cryptosporidium* spp. sub-families, stool samples from Ghanaian PLWH harbored DNA of *C. hominis* Ib, *C. hominis* Ie, and *C. parvum* IIc at similar frequencies, followed by *C. hominis* Ia, *C. hominis* Id, and *C. parvum* IIe at lower frequencies. Notably, *C. parvum* IIc exhibited the highest detection rate among participants with CD4+ T cell counts >200/µL and was the only sub-family detected more than once in this subgroup.

Compared to a previous study in Ghanaian children without known HIV infection during a similar period, the most frequently observed sub-families (*C. parvum* IIc, *C. hominis* Ia, and *C. hominis* Ib) were consistent [23]. The other detected sub-families, *C. hominis* Id, *C. hominis* Ie, and *C. parvum* IIc, also matched previous findings [23]. These observations suggest a relatively stable distribution of these six *Cryptospordium* spp. sub-families in Ghana regardless of the immunological status or population studied. Even though the order of prevalence differed, *C. hominis* Ib and *C. parvum* IIc were among the top three in both Ghanaian PLWH and children [23].

Despite similarities, notable peculiarities were observed in the PLWH population. Specifically, while most *Cryptosporidium* spp. detections occurred in individuals with low CD4+ T cell counts, *C. parvum* IIc was most frequently detected among PLWH with CD4+ T cell counts above 200/µL. This parallel with findings in Ghanaian children [23] suggests that *C. parvum* IIc may be less dependent on host immunosuppression compared to other *Cryptosporidium* spp. sub-families. This hypothesis aligns with the multivariable regression results, which revealed significant associations between *C. hominis* sub-families and reduced CD4+ T cell counts, but not for *C. parvum* IIc or IIe. Additionally, similarly high levels of anthroponotic *C. hominis* IIc circulation, irrespective of HIV status, have been reported in West African Nigeria [37]. In severely immunocompromised individuals, *C. parvum* IIc infection has been linked to severe cryptosporidiosis [38], suggesting a potential for increased virulence. Conversely, *C. hominis* Ia and Ib sub-families were more frequent among HIV-positive individuals not receiving anti-retroviral therapy, with *C. hominis* Ia especially common in recently diagnosed cases. Together with their significant associations with reduced CD4+ counts, these findings support the hypothesis that these sub-families thrive under severe immunosuppression. Future studies should evaluate whether cluster-specific detection of *C. parvum* IIc warrants intensified infection prevention and control measures, given its persistence at higher CD4+ levels and possible altered virulence or transmission dynamics.

The distribution of *Cryptosporidium* spp. sub-families among PLWH appears remarkably consistent across West African countries, with similar profiles reported in Nigeria and Equatorial Guinea [39,40]. Even in East African Ethiopia, the sub-family distribution was comparable [41].

Regardless of sub-family, *Cryptosporidium* spp. preferentially occurred in newly diagnosed PLWH with CD4+ T cell counts <200/µL, a population at particular risk. This emphasizes the need to explore low-level diagnostic screening for *Cryptosporidium* spp. in such immunocompromised patients.

Epidemiological data on *Cryptosporidium* sub-family distributions may prove valuable for future therapeutic development. Current comprehensive reviews [42,43] highlight ongoing efforts to expand treatment beyond immune restoration [4]. While sub-family-specific therapies remain distant [42,43], differential drug effects could influence future therapeutic strategies.

Regarding *Giardia duodenalis*, no significant associations with HIV infection or any specific assemblage were detected in this study. The observed trend linking lack of tap water access to giardiasis reaffirms established fecal–oral transmission routes [44]. A non-significant tendency associating assemblage B and non-A-non-B assemblages with skin rash was a likely statistical artifact. These results support the critical view questioning a strong HIV–giardiasis link [11]. Notably, non-significant trends suggested a reduced CD4+/CD8+ ratio and increased *G. duodenalis* quantity (by Ct value) associated with low CD4+ cell counts and high HIV viral loads, paralleling a previous US study indicating trends toward reduced CD4+ T-lymphocytes in HIV patients co-infected with *G. duodenalis* [13]. However, the lack of statistical significance tempers interpretation.

This study has a number of limitations. First and most importantly, the retrospective design precluded sample size adjustments based on expected effect sizes, potentially missing small associations. The associated very small subgroup sizes reduced the statistical power of the study. Second, limited epidemiologic data constrained analytical options. Third, interpretable *Cryptospodium* spp. sequencing was possible in only a minority of PCR-positive samples, with some sequences short and low quality, necessitating cautious interpretation. Therefore, phylogenetic analysis beyond the sub-family level was avoided to prevent erroneous conclusions.

## 5. Conclusions

This study confirmed a strong association between cryptosporidiosis and HIV infection in the Ghanaian cohort examined. DNA of *C. hominis* Ib, *C. hominis* Ie, and *C. parvum* IIc were detected at similar frequencies, followed by *C. hominis* Ia and *C. hominis* Id, and *C. parvum* IIe at lower frequencies. Notably, only *C. parvum* IIc was repeatedly detected in individuals with CD4+ T cell counts greater than 200/µL. *C. hominis* Ia and Ib were associated with PLWH not receiving ART, with *C. hominis* Ia additionally linked to recently diagnosed HIV infections. These findings suggest the preferential occurrence of certain sub-families in individuals with severe immunosuppression. In contrast, no significant associations were found between *G. duodenalis* assemblages and HIV infection, indicating that other factors likely influence *G. duodenalis* epidemiology in Ghana. Future research should focus on longitudinal studies to track infection dynamics relative to cART initiation and evaluate the integration of both traditional and molecular parasitic diagnostics into routine HIV care in endemic settings. Particularly for resource-limited areas, there is a critical need to develop and implement affordable, field-adapted diagnostic tools.

## Figures and Tables

**Table 1:** Assignment of samples to *Cryptosporidium* spp. sub-families and *G. duodenalis* assemblages. The assessment included 905 stool samples from 730 Ghanaian patients with and without HIV infection.

Step	*Cryptosporidium* spp.	*G. duodenalis*
Screening for positive PCR signals	114 samples positive in ≥1/3 screening PCRs	123 samples positive in ≥1/3 screening PCRs
Exclusion due to insufficient sequence data	67 excluded: failed/insufficient nested PCR	97 excluded: only 1/3 PCRs positive (33 in PCR 1, 64 in PCR 3)
Exclusion of duplicate patient samples	5 excluded as duplicates in follow-up samples	None
Final assignment to sub-families/assemblages	*C. hominis*: Ia (n = 4), Ib (n = 15), Id (n = 3), Ie (n = 8) *C. parvum*: IIc (n = 8), IIe (n = 3)	Assemblage A (n = 6); assemblage B (n = 12); non-A-non-B (n = 8)
Assignment details and exclusions	Sequence assignments detailed in Appendix A Table A5	False-positive assemblage signals excluded: B PCR 2 (n = 2), A PCR 2 (n = 1), B PCR 3 (n = 1)
Co-infections	Co-occurrence of *G. duodenalis* and *Cryptosporidium* spp. very rare; specifically, *G. duodenalis* assemblage B with *C. hominis* Ib (1 sample), and *G. duodenalis* assemblage B with *C. parvum* IIc (1 sample). No obvious clustering observed.	

PCR: polymerase chain reaction; numbers in parentheses indicate sample count and specific cluster or assemblage assigned; all exclusion/inclusion criteria as described in the Methods; Appendix A tables provide detailed primer and sequencing information.

**Table 2:** Demographics, socio-economic parameters, medical treatment, and clinical parameters in HIV-positive participants according to the detection of *Cryptosporidium* spp. sub-families.

Variable	No *Cryptosporidium* spp.	Ia	Ib	Id	Ie	IIc	IIe	*p*-Value	FDR-Corrected Pairwise *p*-Values
Age in years, median (IQR)	40 (34/47)	38 (32/50)	39 (30/44)	50 (32/52)	38 (32/47)	33 (27/50)	40 (32/48)	0.889	
Female, n (%)	436 (73.52)	3 (75)	14 (93.33)	1 (33.33)	4 (50)	6 (75)	1 (50)	0.121	
Access to tap water, n (%)	318 (53.63)	1 (25)	5 (33.33)	1 (33.33)	5 (62.5)	5 (62.5)	0 (0)	0.341	
Electricity in household, n (%)	555 (93.59)	4 (100)	14 (93.33)	3 (100)	8 (100)	7 (87.5)	1 (50)	0.325	
Refrigerator in household, n (%)	427 (72.01)	4 (100)	13 (86.67)	2 (66.67)	7 (87.5)	7 (87.5)	1 (50)	0.524	
Intake of cART, n (%)	260 (43.48)	0 (0)	0 (0)	1 (33.33)	0 (0)	2 (25)	0 (0)	0.001	b
TMP/SMX prophylaxis, n (%)	189 (32.53)	0 (0)	6 (42.86)	2 (66.67)	3 (37.5)	6 (75)	0 (0)	0.067	
Body mass index, median (IQR)	22 (20/26)	23 (22/24)	22 (19/29)	21 (19/22)	20 (20/21)	22 (20/24)	23 (20/27)	0.527	
Days since HIV diagnosis, median (IQR)	296 (12/1760)	12 (2/24)	14 (11/26)	19 (8/2228)	20 (8/101)	49 (10/168)	0 (0/0)	0.005	b
Cough *, n (%)	62 (10.46)	0 (0)	5 (33.33)	1 (33.33)	0 (0)	3 (37.5)	1 (50)	0.006	n.s.
Diarrhea *, n (%)	35 (5.9)	2 (50)	1 (6.67)	0 (0)	3 (37.5)	1 (12.5)	0 (0)	0.007	n.s.
Fever *, n (%)	34 (8.19)	1 (33.33)	3 (30)	0 (0)	3 (60)	0 (0)	0 (0)	0.005	n.s.
Skin rash *, n (%)	41 (6.91)	2 (50)	3 (20)	0 (0)	3 (37.5)	0 (0)	1 (50)	0.001	n.s.
Weight loss *, n (%)	132 (22.26)	3 (75)	8 (53.33)	2 (66.67)	4 (50)	4 (50)	1 (50)	0.001	n.s.

FDR—false discovery rate; TMP/SMX—trimethoprim/sulfamethoxazole; IQR—interquartile range; cART—antiretroviral combination therapy; * self-reported clinical symptoms that occurred during the last 6 months. FDR-corrected pairwise *p*-values indicate statistically significant differences between the control group and each *Cryptosporidium* sub-family. Specifically, “b” denotes the comparison with sub-family Ib. “n.s.” indicates no significant comparison.

**Table 3:** Demographics, socio-economic, medical-treatment-related, and clinical parameters in HIV-positive participants according to the detection of *G. duodenalis* assemblages.

Variable	No *G. duodenalis*	Assemblage A	Assemblage B	Non-A-non-B Assemblage	*p*-Value	FDR-Corrected *p*-Values
Age in years, median (IQR)	40 (33/48)	38 (33/42)	34 (31/39)	46 (39/51)	0.153	
Female, n (%)	449 (73.25)	4 (80)	9 (100)	3 (50)	0.124	
Access to tap water, n (%)	325 (53.02)	1 (20)	3 (33.33)	6 (100)	0.025	n.s.
Electricity in household, n (%)	572 (93.31)	5 (100)	9 (100)	6 (100)	1.000	
Refrigerator in household, n (%)	443 (72.27)	5 (100)	7 (77.78)	6 (100)	0.324	
Intake of cART, n (%)	254 (41.44)	3 (60)	3 (33.33)	3 (50)	0.756	
TMP/SMX prophylaxis, n (%)	196 (32.67)	2 (40)	4 (44.44)	4 (66.67)	0.241	
Body mass index, median (IQR)	22 (20/26)	23 (20/29)	24 (22/26)	21 (20/27)	0.678	
Days since HIV diagnosis, median (IQR)	186 (12/1701)	1258 (530/2195)	78 (16/2113)	28 (5/1361)	0.497	
Cough *, n (%)	68 (11.09)	1 (20)	2 (22.22)	1 (16.67)	0.318	
Diarrhea *, n (%)	40 (6.53)	0 (0)	1 (11.11)	1 (16.67)	0.396	
Fever *, n (%)	39 (9.20)	0 (0)	0 (0)	2 (40)	0.165	
Skin rash *, n (%)	45 (7.34)	0 (0)	3 (33.33)	2 (33.33)	0.012	n.s.
Weight loss *, n (%)	150 (24.47)	1 (20)	2 (22.22)	1 (16.67)	1.000	

FDR—false discovery rate; TMP/SMX—trimethoprim/sulfamethoxazole; IQR—interquartile range; cART—antiretroviral combination therapy; * self-reported clinical symptoms that occurred during the last 6 months. FDR-corrected pairwise *p*-values indicate statistically significant differences between the “No *G. duodenalis*” group (without detectable *G. duodenalis* DNA) and each assemblage. “n.s.” indicates non-significant comparison.

**Table 4:** Virological and immunological parameters according to *Cryptosporidium* spp. sub-families.

Variable	No *Cryptosporidium* spp.	Ia	Ib	Id	Ie	IIc	IIe	*p*-Value	FDR-Corrected *p*-Values
Viral load, log10 copies/mL, median (IQR)	4.1 (1.6/5.3)	5.6 (5.2/6.0)	5.7 (5.2/5.9)	6.6 (6.3/7.0)	5.5 (5.4/5.8)	5.2 (4.5/5.5)	5.3 (4.9/5.7)	<0.001	a, b, c, d, e
CD4+ T cell count/µL, median (IQR)	359 (186/589)	42 (12/71)	60 (16/99)	53 (51/88)	40 (20/63)	155 (40/291)	24 (19/30)	<0.001	a, b, c, d, e, f
CD8+ T cell count/µL, median (IQR)	984 (671/1384)	984 (336/2251)	577 (413/868)	1115 (818/2262)	447 (341/1577)	808 (175/1922)	984 (840/1127)	0.307	
CD4+/CD8+ T cell ratio, median (IQR)	0.39 (0.19/0.69)	0.04 (0.01/0.06)	0.12 (0.06/0.18)	0.05 (0.03/0.11)	0.06 (0.02/0.15)	0.11 (0.08/0.16)	0.02 (0.02/0.03)	<0.001	a, b, c, d, e, f

FDR—false discovery rate; IQR—interquartile range. FDR-corrected pairwise *p*-values indicate statistically significant differences between the control group and each *Cryptosporidium* spp. sub-family. Specifically, “a” denotes the comparison with sub-family Ia, “b” with Ib, “c” with Id, “d” with Ie, “e” with IIc, and “f” with IIe.

**Table 5:** Virological and immunological parameters according to *G. duodenalis* assemblages.

Variable	No *G. duodenalis*	Assemblage A	Assemblage B	Non-A-non-B Assemblage	*p*-Value
Viral load, log10 copies/mL, median (IQR)	4.3 (1.6/5.4)	1.6 (0.3/3.6)	4.2 (2.4/5.3)	3.2 (1.5/5.1)	0.556
CD4+ T cell count/µL, median (IQR)	348 (152/572)	345 (274/672)	116 (8/411)	265 (52/441)	0.153
CD8+ T cell count/µL, median (IQR)	974 (654/1371)	955 (286/1753)	1002 (413/1580)	747 (591/1114)	0.915
CD4+/CD8+ T cell ratio, median (IQR)	0.4 (0.2/0.7)	0.7 (0.2/2.2)	0.1 (0.0/0.3)	0.5 (0.4/0.6)	0.067

IQR—Interquartile range.

**Table 6:** Correlation of cycle threshold (Ct) values of the real-time PCR targeting *G. duodenalis* and its assemblages with immunological and virological parameters in the entire cohort.

	Ct Values of the Real-Time PCR Targeting *G. duodenalis*		Ct Values of the Real-Time PCR Targeting *G. duodenalis* Assemblage A		Ct Values of the Real-Time PCR Targeting *G. duodenalis* Assemblage B	
	Kendall’s Tau	*p*-Value	Kendall’s Tau	*p*-Value	Kendall’s Tau	*p*-Value
CD4+ T cell count/µL	0.27	0.112	0.26	0.513	0.22	0.392
CD4+/CD8+ T cell ratio	0.32	0.103	−0.26	0.564	0.22	0.469
Viral load, log10 copies/mL	−0.25	0.227	−0.82	0.221	−0.31	0.301

The Ct values for the assemblages were defined by selecting the lowest Ct value from all PCR measurements per sample and categorizing them into three ordinal groups: low Ct (<25), medium Ct (25–35), and high Ct (>35); correlation analysis was performed using these categories.

## Data Availability

All relevant data are provided in the manuscript or its Appendix A tables. Raw data can be made available upon reasonable request.

## Abbreviations

The following abbreviations are used in this manuscript:

95% CI	95% confidence interval
AIDS	acquired immune deficiency syndrome
cART	combined anti-retroviral therapy
Ct	cycle threshold
DNA	deoxyribonucleic acid
FDR	false discovery rate
HIV	human immunodeficiency virus
IQR	interquartile range
Min.	minute
MSM	men who have sex with men
N	number
n.e.	not estimable
OR	odds ratio
PBMC	peripheral blood mononuclear cells
PCR	polymerase chain reaction
PLWH	people living with HIV
SD	standard deviation
Sec.	second
TMP/SMX	trimethoprim/sulfamethoxazole

## Appendix A

**Table A1:** Oligonucleotides used for the *Cryptosporidium* spp.-specific nested PCR and for the subsequent Sanger sequencing. Hyphens in the oligonucleotide sequences have been inserted to increase the readability, not to delineate codon triplets.

PCR target	*Cryptosporidium* spp. (primary PCR)
Target gene	GP60 gene
Forward primer	5′-ATA-GTC-TCC-GCT-GTA-TTC-3′
Reverse primer	5′-GGA-AGG-AAC-GAT-GTA-TCT-3′
Positive control plasmid insert	5′-ACT-CTC-CGT-TAT-AGT-CTC-CGC-TGT-ATT-CTC-AGC-CCC-AGC-CGT-TCC-ACT-CAG-AGG-AAC-TTT-AAA-GGA-TGT-TCC-TGT-TGA-GGG-CTC-ATC-ATC-GTC-ATC-GTC-ATC-GTC-ATC-GTC-ATC-ATC-ATC-ATC-ATC-ATC-ATC-ATC-ATC-ATC-ATC-AAC-ATC-AAC-CGT-CGC-ACC-AGC-AAA-TAA-GGC-AAG-AAC-TGG-AGA-AGA-CGC-AGA-AGG-CAG-TCA-AGA-TTC-TAG-TGG-TAC-TGA-AGC-TTC-TGG-TAG-CCA-GGG-TTC-TGA-AGA-GGA-AGG-TAG-TGA-AGA-CGA-TGG-CCA-AAC-TAG-TGC-TGC-TTC-CCA-ACC-CAC-TAC-TCC-AGC-TCA-AAG-TGA-AGG-CGC-AAC-TAC-CGA-AAC-CAT-AGA-AGC-TAC-TCC-AAA-AGA-AGA-ATG-CGG-CAC-TTC-ATT-TGT-AAT-GTG-GTT-CGG-AGA-AGG-TAC-CCC-AGC-TGC-GAC-ATT-GAA-GTG-TGG-TGC-CTA-CAC-TAT-CGT-CTA-TGC-ACC-TAT-AAA-AGA-CCA-AAC-AGA-TCC-CGC-ACC-AAG-ATA-TAT-CTC-TGG-TGA-AGT-TAC-ATC-TGT-AAC-CTT-TGA-AAA-GAG-TGA-TAA-TAC-AGT-TAA-AAT-CAA-GGT-TAA-CGG-TCA-GGA-TTT-CAG-CAC-TCT-CTC-TGC-TAA-TTC-AAG-TAG-TCC-AAC-TGA-AAA-TGG-CGG-ATC-TGC-GGG-TCA-GGC-TTC-ATC-AAG-ATC-AAG-AAG-ATC-ACT-CTC-AGA-GGA-AAC-CAG-TGA-AGC-TGC-TGC-AAC-CGT-CGA-TTT-GTT-TGC-CTT-TAC-CCT-TGA-TGG-TGG-TAA-AAG-AAT-TGA-AGT-GGC-TGT-ACC-AAA-CGT-CGA-AGA-TGC-ATC-TAA-AAG-AGA-CAA-GTA-CAG-TTT-GGT-TGC-AGA-CGA-TAA-ACC-TTT-CTA-TAC-CGG-CGC-AAA-CAG-CGG-CAC-TAC-CAA-TGG-TGT-CTA-CAG-GTT-GAA-TGA-GAA-CGG-AGA-CTT-GGT-TGA-TAA-GGA-CAA-CAC-AGT-TCT-TTT-GAA-GGA-TGC-TGG-TTC-CTC-TGC-TTT-TGG-ACT-CAG-ATA-CAT-CGT-TCC-TTC-CGT-TTT-TGC-AA-3′
GenBank accession number used for the insert	MK034696.1
Reference	[21,22]
PCR target	*Cryptosporidium* spp. (secondary PCR)
Target gene	GP60 gene
Forward primer	5′-TCC-GCT-GTA-TTC-TCA-GCC-3′
Reverse primer	5′-GCA-GAG-GAA-CCA-GCA-TC-3′
Reference	[21,22]
Sequencing target	*Cryptosporidium* spp. (Sanger sequencing)
Target gene	GP60 gene
Forward primer	5′-TCC-GCT-GTA-TTC-TCA-GCC-3′
Reverse primer	5′-GCA-GAG-GAA-CCA-GCA-TC-3′
Reference	[21,22]

**Table A2:** Reaction mixes and run conditions for PCR assays targeting *Cryptosporidium* spp.

	*Cryptosporidium* spp. (Primary PCR)	*Cryptosporidium* spp. (Secondary PCR)
Reaction chemistry		
Master Mix	HotStar master mix (Qiagen, Hilden, Germany)	HotStar master mix (Qiagen, Hilden, Germany)
Reaction volume (µL)	25	50
Forward primer concentration (pmol/µL)	40	20
Reverse primer concentration (pmol/µL)	40	20
Final Mg^2+^ concentration (mM)	5	3
Eluate volume (µL)	2	10
Run conditions		
Initial denaturation	10 min. 94 °C	10 min. 94 °C
Cycle numbers	35	35
Denaturation	45 s. 94 °C	45 s. 94 °C
Annealing	45 s. 55 °C	45 s. 55 °C
Amplification	60 s. 72 °C	60 s. 72 °C
Hold Cooling	7 min. 72 °C 30 s. 40 °C	7 min. 72 °C 30 s. 40 °C

min. = minute; s. = second.

**Table A3:** Target genes, calculated detection limits, and oligonucleotides used for the applied real-time PCR screening assays for *Giardia duodenalis*, as well as assemblages A and B. Hyphens in the oligonucleotide sequences have been inserted to increase the readability, not to delineate codon triplets.

PCR target	*Giardia duodenalis* PCR 1
Target gene	*bg*
Detection limit	*<10* copies/µL
Forward primer	5′-CAT-CCG-CGA-GGA-GGT-CAA-3′
Reverse primer	5′-GCA-GCC-ATG-GTG-TCG-ATC-T-3′
Probe and modifications	5′-Cy5-AAG-TCC-GCC-GAC-AAC-ATG-TAC-CTA-ACG-A-BHQ2-3′
Positive control plasmid insert	5′-*CGT-TCG-AGG-ACA-TCC-GCG-AGG-AGG-TCA-AGA-AGT-CCG-CCG-ACA-ACA-TGT-ACC-TAA-CGA-TCA-AGG-AGG-AGA-TCG-ACA-CCA-TGG-CTG-CAA-ACT-TCC-GC*-3′
GenBank accession number used for the insert	KT728533.1
Reference	[25,31]
PCR target	*Giardia duodenalis* PCR 2
Target gene	18S rRNA gene sequence
Detection limit	*<10* copies/µL
Forward primer	5′-GAC-GGC-TCA-GGA-CAA-CGG-TT-3′
Reverse primer	5′-TTG-CCA-GCG-GTG-TCC-G-3′
Probe and modifications	5′-FAM-CCC-GCG-GCG-GTC-CCT-GCT-AG-BHQ1-3′
Positive control plasmid insert	5′-*GGA-CGC-GGC-GGA-CGG-CTC-AGG-ACA-ACG-GTT-GCA-CCC-CCC-GCG-GCG-GTC-CCT-GCT-AGC-CGG-ACA-CCG-CTG-GCA-ACC-CGG-CGC-CA*-3′
GenBank accession number used for the insert	MT484081.1
Reference	[25,32]
PCR target	*Giardia duodenalis* PCR 3
Target gene	*gdh*
Detection limit	*<10* copies/µL
Forward primer	5′-CTG-AAG-AAC-TCC-CTC-ACC-AC-3′
Reverse primer	5′-CAG-AAG-CGC-ATG-ACC-TCG-TTG-3′
Probe and modifications	5′-HEX-CAA-GGG-CGG-CTC-CGA-CTT-TGA-CCC-AA-BHQ1-3′
Positive control plasmid insert	5′-*TCG-AGC-AGA-TCC-TGA-AGA-ACT-CCC-TCA-CCA-CGC-TCC-CGA-TGG-GCG-GCG-GCA-AGG-GCG-GCT-CCG-ACT-TTG-ACC-CAA-AGG-GCA-AGT-CCG-ACA-ACG-AGG-TCA-TGC-GCT-TCT-GCC-AGT-CCT-TC*-3′
GenBank accession number used for the insert	MH311031.1
Reference	[25,33]
PCR target	*Giardia duodenalis* assemblage A PCR 1
Target gene	*Bg*
Detection limit	*<10* copies/µL
Forward primer	5′-CCT-CAA-GAG-CCT-GAA-CGA-TCT-C-3′
Reverse primer	5′-AGC-TGG-TCG-TAC-ATC-TTC-TTC-CTT-3′
Probe and modifications	5′-FAM-TTC-TCC-GTG-GCA-ATG-CCC-GTC-T-BHQ1-3′
Positive control plasmid insert	5′-GGA-AGG-AGG-CCC-TCA-AGA-GCC-TGA-ACG-ATC-TCG-AGA-CGG-GCA-TTG-CCA-CGG-AGA-ACG-CAG-AAA-GGA-AGA-AGA-TGT-ACG-ACC-AGC-TCA-ACG-AGA-AG-3′
GenBank accession number used for the insert	KT728533.1
Reference	[25,31]
PCR target	*Giardia duodenalis* assemblage A PCR 2
Target gene	*bg*
Detection limit	83 copies/µL
Forward primer	5′-CCT-CAA-GAG-CCT-GAA-CGA-TCT-C-3′
Reverse primer	5′-AGC-TGG-TCG-TAC-ATC-TTC-TTC-CTT-3′
Probe and modifications	5′-6-FAM-TGG-C+A+A-TGC-C+CG-+TCT-BHQ1-3′
Positive control plasmid insert	5′-GGA-AGG-AGG-CCC-TCA-AGA-GCC-TGA-ACG-ATC-TCG-AGA-CGG-GCA-TTG-CCA-CGG-AGA-ACG-CAG-AAA-GGA-AGA-AGA-TGT-ACG-ACC-AGC-TCA-ACG-AGA-AG-3′
GenBank accession number used for the insert	KT728533.1
Reference	[25,34]
PCR target	*Giardia duodenalis* assemblage A PCR 3
Target gene	*tpi*
Detection limit	*<10* copies/µL
Forward primer	5′-CAT-TGC-CCC-TTC-CGC-C-3′
Reverse primer	5′-CTG-CGC-TGC-TAT-CCT-CAA-CTG-3′
Probe and modifications	5′-VIC-CCA-TTG-CGG-CAA-ACA-MGB-NFQ-3′
Positive control plasmid insert	5′-TGG-ACG-TCG-TCA-TTG-CCC-CTT-CCG-CCG-TAC-ACC-TGT-CAA-CAG-CCA-TTG-CGG-CAA-ACA-CGT-CAA-AAC-AGT-TGA-GGA-TAG-CAG-CGC-AGA-ATG-TGT-ACC-3′
GenBank accession number used for the insert	MZ822181.1
Reference	[25,35]
PCR target	*Giardia duodenalis* assemblage B PCR 1
Target gene	*bg*
Detection limit	*<10* copies/µL
Forward primer	5′-CCT-CAA-GAG-CCT-GAA-CGA-CCT-C-3′
Reverse primer	5′-AGC-TGG-TCA-TAC-ATC-TTC-TTC-CTC-3′
Probe and modifications	5′-Cy5-TTC-TCC-GTG-GCG-ATG-CCT-GTC-T-BHQ2-3′
Positive control plasmid insert	5′-GGA-AGG-AGG-CCC-TCA-AGA-GCC-TGA-ACG-ACC-TCG-AGA-CAG-GCA-TCG-CCA-CGG-AGA-ACG-CCG-AGA-GGA-AGA-AGA-TGT-ATG-ACC-AGC-TCA-ACG-AGA-AA-3′
GenBank accession number used for the insert	PP566783.1
Reference	[25,31]
PCR target	*Giardia duodenalis* assemblage B PCR 2
Target gene	*bg*
Detection limit	*83* copies/µL
Forward primer	5′-CCT-CAA-GAG-CCT-GAA-CGA-CCT-C-3′
Reverse primer	5′-AGC-TGG-TCA-TAC-ATC-TTC-TTC-CTC-3′
Probe and modifications	5′-Cy5-TGG-CG+A-TGC-+C+T+G-TCT-BHQ2-3′
Positive control plasmid insert	5′-GGA-AGG-AGG-CCC-TCA-AGA-GCC-TGA-ACG-ACC-TCG-AGA-CAG-GCA-TCG-CCA-CGG-AGA-ACG-CCG-AGA-GGA-AGA-AGA-TGT-ATG-ACC-AGC-TCA-ACG-AGA-AA-3′
GenBank accession number used for the insert	PP566783.1
Reference	[25,34]
PCR target	*Giardia duodenalis* assemblage B PCR 3
Target gene	*tpi*
Detection limit	*<10* copies/µL
Forward primer	5′-GAT-GAA-CGC-AAG-GCC-AAT-AA-3′
Reverse primer	5′-TCT-TTG-ATT-CTC-CAA-TCT-CCT-TCT-T-3′
Probe and modifications	5′-FAM-AAT-ATT-GCT-CAG-CTC-GAG-MGB-NFQ-3′
Positive control plasmid insert	5′-AGA-GAC-CCT-GGA-TGA-ACG-CAA-GGC-CAA-TAA-CAC-TAT-GGA-GGT-GAA-TAT-TGC-TCA-GCT-CGA-GGC-TCT-TAA-GAA-GGA-GAT-TGG-AGA-ATC-AAA-GAA-GTT-ATG-GGA-3′
GenBank accession number used for the insert	KU311953.1
Reference	[25,35]

+ = following base is LCA (locked nucleic acid).

**Table A4:** Reaction mixes and run conditions for *Giardia duodenalis*, as well as assemblages A and B.

	*G. duodenalis* PCR 1-3	*G. duodenalis* Assemblage A PCR 1, *G. duodenalis* Assemblage B PCR 1	*G. duodenalis* Assemblage A PCR 2, *G. duodenalis* Assemblage B PCR 2	*G. duodenalis* Assemblage A PCR 3, *G. duodenalis* Assemblage B PCR 3
Reaction chemistry				
Master Mix	HotStar master mix (Qiagen, Hilden, Germany)	HotStar master mix (Qiagen, Hilden, Germany)	HotStar master mix (Qiagen, Hilden, Germany)	HotStar master mix (Qiagen, Hilden, Germany)
Reaction volume (µL)	20.0	20.0	20.0	20.0
Forward primer concentration (nM)	300.0 (PCR 1), 125.0 (PCR 2), 200.0 (PCR 3)	300.0	300.0	300.0
Reverse primer concentration (nM)	300.0 (PCR 1), 125.0 (PCR 2), 200.0 (PCR 3)	300.0	300.0	900.0
Probe concentration (nM)	6.0 (PCR 1), 10.0 (PCR 2), 200.0 (PCR 3)	200.0	200.0	100.0
Final Mg^2+^ concentration (mM)	1.5	4.0	3.0	3.0
Bovine serum albumin (mg/mL)	2.0	2.0	2.0	2.0
Eluate volume (µL)	2.0	2.0	2.0	2.0
Run conditions				
Initial denaturation	15 min. at 95 °C	15 min. at 95 °C	15 min. at 95 °C	15 min. at 95 °C
Cycle numbers	40	40	50	50
Denaturation	15 s. at 95 °C	15 s. at 95 °C	15 s. at 95 °C	15 s. at 95 °C
Annealing	60 s. at 60 °C	60 s. at 60 °C	8 s. at 58 °C	60 s. at 60 °C
Amplification	same as annealing	same as annealing	3 s. at 72 °C	same as annealing
Hold	10 s. at 40 °C	10 s. at 40 °C	10 s. at 40 °C	10 s. at 40 °C

min. = minute; s. = second.

**Table A5:** Sequences of the obtained amplicons. Dashes within the sequences are included to improve readability, not to delineate amino acid–coding base triplets.

Anonymized Sample-ID	Sequence	NCBI GenBank Accession Number of Best Matching Sequence	Species Level Assignment	Sub-Family Level Assignment
1	5′-TTT-TTC-TCA-GCC-CCA-GCC-GTT-CCA-CTC-AGA-GGC-ACT-TTA-AAG-GAT-GTT-TCT-GTT-GAG-AGC-TCA-TCG-TCA-TCA-TCG-TCA-TCG-TCA-ACA-ACC-CCC-GCA-CCA-GCT-CCA-AAG-AAG-GTA-AGA-GAA-AGC-GAA-GAA-GGG-AAG-AAC-AGT-GAA-GAT-AGT-CAA-ACT-CCC-GCT-AGT-CCT-GGA-AGT-GAT-TCT-CAG-GAT-AGC-TCT-AAA-GGA-GAC-GAA-GTT-GTA-GAT-GGA-GGC-GCT-TCC-GGA-TCT-AGT-ACC-CCA-ACT-CAA-GCT-GCT-GAA-AAG-GAG-CCC-GAA-ACT-CCA-GAA-TCT-ACT-CCA-AAG-GAA-GAA-TGT-GGT-ACT-TCA-TTT-ATA-ATG-TGG-TTC-GGA-GAA-GGT-ACT-CCA-GCC-ACA-ACT-TTG-AAG-TGC-GGT-GGC-TAC-ACT-ATC-GTC-TAT-GCA-CCA-GAA-AAG-GAT-AAT-AAA-GAA-CCC-GCA-CCA-AGA-TAC-ATC-TCT-GGT-GAT-GTT-AAG-GCT-GTA-ACC-TTT-GAA-AAG-GGA-GAA-GAT-AAT-ACA-GTT-AAA-ATC-AAG-GTT-GAT-GGT-AAG-GAG-TTC-AGT-ACT-CTC-TCT-TCT-AGC-TCA-AGC-AGT-CCA-ACT-GAA-AAT-AAC-GGA-TCT-ACG-GGC-CAG-GTT-GCA-TCA-AGA-TCA-AGA-AGA-TCG-CTC-TCA-GAG-GAA-AAT-AGT-GAA-ACT-GCT-GCA-ACC-GTC-GAT-TTG-TTT-GCC-TTC-ACC-CTT-CAA-GGT-GGT-AAA-AGA-ATC-GAA-GTC-GCT-GTG-CCA-AGT-GAC-AAA-GAT-GTA-TCC-AAG-AGA-AAC-AAG-TAC-AGT-TTG-GTT-GCA-GGC-GAT-AAG-ACT-TTC-TAT-ACC-GGC-GCA-AAT-AGC-GGT-AAT-ACC-GAC-GGT-ATCT-ACA-GGT-TGA-ATG-ATG-ATG-GAG-ACT-TGG-TGG-ACA-AGA-ACA-ACA-ACG-TTC-TTT-TGA-AGG-ATG-TG-3′	KU670812	*C. parvum*	IIc
2	5′-KTT-TTT-TTT-YAC-CCA-GCC-GTT-CCA-CTC-AGA-GGC-ACT-TTA-AAG-GAT-GCT-TCT-GTT-GAG-GGC-TCA-TCA-TCA-TCA-TCA-TCA-TCA-TCA-TCA-TCA-TCG-ACC-ACC-GTC-GCA-CCA-GCT-CCA-AAG-AAA-GAA-AGA-ACT-GGA-GAG-GGC-GTA-GAT-GGA-AAG-GAC-CAA-GTA-GAT-AGT-ACA-GGT-TCT-GAT-CAG-AAC-AGT-AAA-GGA-GAC-ACT-AAA-GGA-ACC-ACA-GAA-GAT-GGT-AAA-GAG-ACC-GAA-GGT-ACT-GTT-TCC-AAA-CCC-ACT-ACT-CCA-GAT-CAA-GGT-GAG-AGC-GCA-ACT-CCC-GGA-TCC-ACG-GAA-ACT-ACT-CCA-AAG-GAA-GAA-TGC-GGT-ACT-TCA-TTT-GTA-ATG-TGG-TTC-GGA-GAA-GGT-ACC-CCA-GTT-GCG-ACC-TTG-AAG-TGT-GGT-GGT-TAC-ACT-ATC-GTC-TAT-GCA-CCT-GTA-AAG-GAA-CAA-ACA-AAT-CCC-GCA-CCA-AGA-TAT-ATC-TCT-GGT-GAG-GTA-AAA-AAT-GTA-TCC-TTC-CAA-AAA-GAA-AGT-GAT-GGT-ACA-ATT-AAAA-TCA-AGA-TTG-ACG-AAA-AGG-AGT-TCA-GTT-CTC-TCT-CTA-CTG-ACT-CAA-GCA-CTC-CAA-CTG-CAA-ATA-GCG-GAT-CCG-CGG-AAC-AGG-TTC-AAT-CAA-GAT-CAA-GAA-GAT-CAC-TCA-CAG-AGG-GAA-GTG-AAA-CAC-CTG-CAA-CCG-TCG-ATT-TGT-TTG-CCT-TCA-CCC-TTG-ATG-GTG-GTA-AAA-GAA-TTG-AAG-TGG-CTG-TAC-CAA-ACA-ACG-AGG-ATG-CAT-CCA-AAA-GAA-CCG-AGT-ACA-GTT-TGG-TTG-CAA-ACG-ATA-AGC-CTT-TCT-ATA-CCG-GCG-CAA-ATA-GCG-GCA-CCG-AAA-ATG-GTG-TCT-ACA-AGT-TGA-ATG-AGA-ACG-GAG-ACT-TGG-TTG-ACA-AGG-ACA-ATA-AAG-TTC-TTT-TGA-ARS-GG-3′	MW480843	*C. parvum*	IIe
3	5′-TTT-TTT-TTY-ACC-CAC-CGT-CCC-ACT-CAG-AGG-CAC-CTT-GAA-GGA-TGT-TTC-TGT-TGA-GGG-CTC-ATC-ATC-ATC-ATC-TTC-ATC-ATC-GTC-TTC-ATC-TTC-ATC-ATC-ATC-ATC-ATC-GTC-AAC-AAC-CCC-AGC-ACC-AGC-TTC-AAA-GAA-GGT-AAG-AGA-AGC-AGA-AGG-CAG-TGT-AGA-AAA-GGG-CAG-TGA-AGA-AAA-GGA-CAG-TGA-AGA-AAA-GGG-CAG-TGA-AGA-AAA-GGG-CAG-TGA-AGA-AGA-TAG-CCA-AAC-TCC-CGC-TAG-TCC-TGG-AGG-TGG-AGG-GGT-GAG-TGA-AGG-AGA-TAC-TCA-AGG-TGA-CTC-TAA-AGG-AGA-CGG-AGT-TAG-TGA-AGA-TGA-GAA-CCA-AAG-TCA-AGG-TGG-GGA-CGC-TAC-TTC-CGA-ATC-TAG-CAC-CCA-AAC-TCA-AGC-TAC-TGA-AAA-AGA-ACC-CGG-ATC-TTC-AGA-AGC-TAC-TCC-AAA-GGA-AGA-GTG-CGG-TAC-TTC-ATT-TGT-AAT-GTG-GTT-CGG-ACA-GGG-TGT-TCC-AGT-TGT-AAC-TTT-GAA-GTG-TGG-TGG-CTA-TAC-TAT-GGT-CTA-TGC-ACC-AGA-AAA-TGG-CAA-AAC-AGA-TCC-CGC-ACC-AAG-ATA-TAT-CTC-TGG-TGA-AGT-TTC-AAC-CGT-AAA-CTT-TGA-AAA-ACA-AGA-TAG-TAC-AGT-TAA-AAT-CAA-GGT-TAA-TGA-TGT-GGA-GTT-CAG-CAC-TCT-CTC-TAC-TAG-CTC-AAG-TAA-TCC-AAC-TGA-AAA-TAG-CGG-ATC-TGA-GAG-CCA-GGC-TCA-ATC-AAG-ATC-AAG-AAG-ATC-ACT-CGC-AGA-GGA-TGG-TAC-TGA-GAC-TGC-TGC-AAC-CGT-CGA-TTT-GAT-TGC-CTT-CAC-CCT-TCA-AGG-TGG-TAA-AAG-AAT-CGA-AGT-CGC-TGT-GCC-AAG-TGA-CGA-AGA-TGC-AGA-CAA-AAG-AAG-CAA-GTA-CAG-TTT-GGT-TGC-AGA-CGA-TAA-GCC-TTT-CTA-TAC-CGG-TGC-AAA-CAG-TGG-AGC-CAC-TGA-TGG-TGT-CTA-CAA-ATT-GGA-TGA-TAA-TGG-AAA-CTT-GGT-AGA-TAA-GGA-CAA-CAA-CGT-TCT-TTG-AGW-GGT-3′	JF727754	*C. hominis*	Ie
4	5′-CTG-TTG-AGG-RAT-CAT-CAT-CAT-CAT-CAT-CAT-CAT-CAT-CAT-CAT-CAT-CAT-CAT-CAT-CAT-CAT-CAT-CAT-CAT-CAA-CAT-CGA-CCG-TCG-CAC-CAG-CTC-CAA-AGA-AAG-AAA-GAA-CTG-TAG-AGG-GCG-GCA-CGG-AAG-GAA-AGA-ACG-AAG-AAA-GCA-GTC-CAG-GTT-CTG-AAG-AAC-AAG-ACG-GTG-GTA-AGG-AGG-ACG-GTG-GTA-AGG-AAA-ACG-GTG-AAG-GAG-ACA-CAG-TAG-ACG-GGG-AAC-AAA-CCG-GGA-GTG-GTT-CTC-AAG-TTA-CTC-CAT-CTG-GAA-GTG-CCG-GCA-CAG-CTA-CCG-AGT-CCA-CAG-CAA-CTA-CTA-CTC-CAA-AGG-AAG-AAT-GTG-GTA-CTT-CAT-TTG-TCA-TGT-GGT-TCG-AGA-AAG-GCA-CCC-CGG-TTG-CGA-CCT-TGA-AGT-GTG-GTG-ATT-ACA-CTA-TCG-TCT-ATG-CAC-CTA-TAA-AAG-ATC-AAA-CAG-ATC-CCG-CAC-CAA-GAT-ATA-TCT-CTG-GTG-AAG-TTA-CAT-CTG-TAT-CCT-TTG-AAA-AGA-GTG-AAA-GTA-CAG-TTA-CAA-TCA-AGG-TTA-ATG-GTA-AAG-AGT-TCA-GCA-CTC-TCT-CTG-CTA-ACT-CAA-GTA-GTC-CAA-CTA-AAG-ATA-ACG-GTG-AAT-CTA-GTG-ACA-GTC-AGG-TTC-AAT-CAA-GAT-CAA-GAA-GAT-CAC-TCG-CAG-AGG-AGA-ATG-GTG-AAA-CAG-TTG-CAA-CAG-TTG-ATT-TGT-TTG-CCT-TTA-CTC-TTG-ATG-GTG-GTA-GAA-GAA-TTG-AAG-TGG-CTG-TGC-CAA-AGG-ACG-AAA-ATG-CAG-ACA-AAA-GAA-GCG-AGT-ACA-GTT-TGG-TTG-CAG-ACG-ATA-AGC-CTT-TCT-ATA-CCG-GCG-CAA-ACA-GTG-GCA-TCA-CCA-ATG-GTG-TCT-ACA-AAT-TGG-ATG-AGA-ATG-GAA-ACT-TGG-TG-3′	KU727289	*C. hominis*	Ia
5	5′-TTT-CTC-AGC-CCC-AGC-CCG-TTC-CAC-TCA-GAG-GCA-CTT-TAA-AGG-ATG-TTT-CTG-TTG-AGA-GCT-CAT-CGT-CAT-CAT-CGT-CAT-CGT-CAA-CAA-CCC-CCG-CAC-CAG-CTC-CAA-AGA-AGG-TAA-GAG-AAA-GCG-AAG-AAG-GGA-AGA-ACA-GTG-AAG-ATA-GTC-AAA-CTC-CCG-CTA-GTC-CTG-GAA-GTG-GTT-CTC-AGG-ATA-GCT-CTA-AAG-GAG-ACG-AAG-TTG-TAG-ATG-GAG-GCGT-TTC-CGG-ATC-TAG-TAC-CCC-AAC-TCA-AGC-TGC-TGA-AAA-GGA-GCC-CGA-AAC-TCC-AGA-ATC-TAC-TCC-AAA-GGA-AGA-ATG-TGG-TAC-TTC-ATT-TAT-AAT-GTG-GTT-CGG-AGA-AGG-TAC-TCC-AGC-CAC-AAC-TTT-GAA-GTG-CGG-TGG-CTA-CAC-TAT-CGT-CTA-TGC-ACC-AGA-AAA-GGA-TAA-TAA-AGA-ACC-CGC-ACC-AAG-ATA-CAT-CTC-TGG-TGA-TGT-TAA-GGC-TGT-AAC-CTT-TGA-AAA-GGG-AGA-AGA-TAA-TAC-AGT-TAA-AAT-CAA-GGT-TGA-TGG-TAG-GGA-GTT-CAG-TAC-TCT-CTC-TTC-TAG-CTC-AAG-CAG-TCC-AAC-TGA-AAA-TAG-CGG-ATC-TGA-GAG-CCA-GGC-TCA-ATC-AAG-ATC-AAG-AAG-ATC-ACT-CAC-AGA-GGG-AAG-TGA-AAC-ACC-TGC-AAC-CGT-CGA-TTT-GTT-TGC-CTT-CAC-CCT-TCA-AGG-TGG-TAA-AAG-AAT-CGA-AGT-CGC-TGT-GCC-AAG-TGA-CGT-AGA-TGC-ATC-CAA-GAG-AAA-CAA-GTA-CAG-TTT-GGT-TGC-AGG-CGA-TAA-GAC-TTT-CTA-TAC-CGG-CGC-AAA-TAG-CGG-TAA-TAC-CGA-CGG-TAT-CTA-CAG-GTT-GAA-TGA-TGA-TGG-AGA-CTT-GGT-GGA-CAA-GAA-CAA-CAA-CGT-TCT-TTT-GAA-GGW-GGK-G-3′	JF802124	*C. parvum*	IIc
6	5′-TTC-CGC-TGY-AMT-CTC-AGC-CCC-AGC-CGT-TCC-ACT-CAG-AGG-CAC-TTT-AAA-GGA-TGT-TTC-TGT-TGA-GAG-CTC-ATC-GTC-ATC-ATY-GTC-ATC-GTC-AAC-AAC-AAC-CCC-CGC-ACC-AGC-TCC-AAA-GAA-GGT-AAG-AGA-AAG-CGA-AGA-AGG-GAA-GAA-CAG-TGA-AGA-TAG-TCA-GAC-TCC-CGC-TAG-TCC-TGG-AAG-TGA-TTC-TCA-GGA-TAG-CTC-TAA-AGG-AGA-CGA-AGC-TGT-AGA-TGG-ATC-CGC-TTC-CGG-ATC-TAG-TAC-CCC-AAC-TCA-AGC-TGC-TGA-AAA-GGA-GCC-CGA-AAC-TCC-AGA-ATC-TAC-TCC-AAA-GGA-AGA-ATG-TGG-TAC-TTC-ATT-TAT-AAT-GTG-GTT-CGG-AGA-AGG-TAC-TCC-AGC-CAC-AAC-TTT-GAA-GTG-CGG-TGG-CTA-CAC-TAT-CGT-CTA-TGC-ACC-AGA-AAA-GGA-TAA-TAA-AGA-ACC-CGC-ACC-AAG-ATA-CAT-CTC-TGG-TGA-TGT-TAA-GGC-TGT-AAC-CTT-TGA-AAA-GGG-AGA-AGA-TAA-TAC-AGT-TAA-AAT-CAA-GGT-TGA-TGG-TAA-GGA-GTT-CAG-TAC-TCT-CTC-TTC-TAG-CTC-AAG-CAG-TCC-AAC-TGA-AAA-TAA-CGG-ATC-TAC-GGG-CCA-GGT-TGC-ATC-AAG-ATC-AAG-AAG-ATC-GCT-CTC-AGA-GGA-AAA-TAG-TGA-AAC-TGC-TGC-AAC-CGT-CGA-TTT-GTT-TGC-CTT-CAC-CCT-TGA-TGG-TGG-CCG-AAG-AAT-TGA-AGT-TGC-TGT-ACC-CAG-CGT-CGA-AGA-TGC-AAC-CAA-AAG-AGA-CAA-GTA-CAG-TTT-GGT-TGC-AAA-CGG-TAA-GCC-TTT-CTA-TAC-CGG-CGC-AAA-CAG-CGG-CAC-TAC-CAA-TGG-TGT-CTA-CAG-GTT-GAA-TGA-GAA-CGG-AGA-CTT-GGT-TGA-CAA-GGA-CAA-CAC-AGT-TCT-CTT-GAA-GGA-TGC-TGG-TTC-CTC-TGC-AAT-T-3′	GU214366	*C. parvum*	IIc
7	5′-KWW-TTT-TCA-GCC-CCA-GCC-GTC-CCA-CTC-AGA-GGC-ACC-TTG-AAG-GAT-GTT-TCT-GTT-GAG-GGC-TCA-TCA-TCA-TCT-TCA-TCA-TCG-TCT-TCA-TCT-TCA-TCA-TCA-TCA-TCG-TCG-TCA-ACA-ACC-CCA-GCA-CCA-GCT-TCA-AAG-AAG-GTA-AGA-GAA-GCA-GAA-GGC-AGT-GAA-GAA-AAG-GAC-AGC-GAA-GAA-AAG-GAC-AGT-GAA-GAA-AAG-GGC-AGT-GAA-GAA-GGT-AGC-CAA-ACT-CCC-GCT-AGT-CCT-GGA-GGT-GGA-GGG-GTG-AGT-GAA-GGA-GAT-ACT-CAA-GGT-GAC-TCT-AAA-GGA-GAC-GGA-GTT-AGT-TCA-GAT-GAG-AAC-CAA-AGT-CAA-GGT-GGG-GAC-GCT-ACT-CCC-GGA-TCT-AGC-ACC-CAA-ACT-CAA-GCT-ACT-GAA-AAA-GAA-CCC-GGA-TCT-TCA-GAA-GCT-ACT-CCA-AAG-GAA-GAG-TGC-GGT-ACT-TCA-TTT-GTA-ATG-TGG-TTC-GGA-CAG-GGT-GTT-CCA-GTT-GTA-ACT-TTG-AAG-TGT-GGT-GGT-TAT-ACT-ATG-GTC-TAT-GCA-CCA-GAA-AAT-GGC-AAA-ACA-GAT-CCC-GCA-CCA-AGA-TAT-ATC-TCT-GGT-AAA-GTT-TCA-ACC-GTA-GAC-TTT-GAA-AAA-CAA-GAT-AGT-ACA-GTT-AAA-ATC-AAG-GTT-AAT-GGT-GTG-GAG-TTC-AGC-ACT-CTC-TCT-ACT-AGC-TCA-AGT-AAT-CCA-ACT-GAA-AAT-AGC-GGA-TCT-GAG-AGC-CAG-GCT-CAA-TCA-AGA-TCA-AGA-AGA-TCA-CTC-GCA-GAG-GAT-GGT-ACT-GAG-ACT-GCT-GCA-ACC-GTC-GAT-TTG-ATT-GCC-TTC-ACC-CTT-CAA-GGT-GGT-AAA-AGA-ATC-GAA-GTC-GCT-GTG-CCA-AGT-GAC-GAA-GAT-GTA-TCC-AAG-AGA-AAC-AAG-TAC-AGT-TTG-GTT-GCA-GGC-GAT-AAG-ACT-TTC-TAT-ACC-GGC-GCA-AAT-AGC-GGT-AAT-ACC-GAC-GGT-ATC-TAC-AGG-TTG-AAT-GAT-GAT-GGA-GAC-TTG-GTG-GAC-AAG-AAC-AAC-AAC-GTT-CTT-TTG-AAG-GAT-GTG-KGK-T-3′	ON863593	*C. hominis*	Ie
8	5′-TTT-TTT-ACC-CCA-GCC-GTT-CCA-CTC-AGA-GGC-ACT-TTA-AAG-GAT-GTT-TCT-GTT-GAG-AGC-TCA-TCG-TCA-TCA-TCG-TCA-TCG-TCA-ACA-ACA-ACC-CCC-GCA-CCA-GCT-CCA-AAG-AAG-GTA-AGA-GAA-AGC-GAA-GAA-GGG-AAG-AAC-AGT-GAA-GAT-AGT-CAG-ACT-CCC-GCT-AGT-CCT-GGA-AGT-GAT-TCT-CAG-GAT-AGC-TCT-AAA-GGA-GAC-GAA-GCT-GTA-GAT-GGA-TCC-GCT-TCC-GGA-TCT-AGT-ACC-CCA-ACT-CAA-GCT-GCT-GAA-AAG-GAG-CCC-GAA-ACT-CCA-GAA-TCT-ACT-CCA-AAG-GAA-GAA-TGT-GGT-ACT-TCA-TTT-ATA-ATG-TGG-TTC-GGA-GAA-GGT-ACT-CCA-GCC-ACA-ACT-TTG-AAG-TGC-GGT-GGC-TAC-ACT-ATC-GTC-TAT-GCA-CCA-GAA-AAG-GAT-AAT-AAA-GAA-CCC-GCA-CCA-AGA-TAC-ATC-TCT-GGT-GAT-GTT-AAG-GCT-GTA-ACC-TTT-GAA-AAG-GGA-GAA-GAT-AAT-ACA-GTT-AAA-ATC-AAG-GTT-GAT-GGT-AAG-GAG-TTC-AGT-ACT-CTC-TCT-TCT-AGC-TCA-AGC-AGT-CCA-ACT-GAA-AAT-AAC-GGA-TCT-ACG-GGC-CAG-GTT-GCA-TCA-AGA-TCA-AGA-AGA-TCG-CTC-TCA-GAG-GAA-AAT-AGT-GAA-ACT-GCT-GCA-ACC-GTC-GAT-TTG-TTT-GCC-TTC-ACC-CTT-GAT-GGT-GGC-CGA-AGA-ATT-GAA-GTT-GCT-GTA-CCC-AGC-GTC-GAA-GAT-GCA-ACC-AAA-AGA-GAC-AAG-TAC-AGT-TTG-GTT-GCA-AAC-GGT-AAG-CCT-TTC-TAT-ACC-GGC-GCA-AAC-AGC-GGC-ACT-ACC-AAT-GGT-GTC-TAC-AGG-TTG-AAT-GAG-AAC-GGA-GAC-TTG-GTT-GAC-AAG-GAC-AAC-ACA-GTT-CTC-TTG-AAG-GWW-Y-3′	MW480840	*C. parvum*	IIc
9	5′-TTT-YYA-GCC-CCA-GCC-GTT-CCA-CTC-AGA-GGC-ACC-TTG-AAA-GAT-GTT-TCT-GTT-GAG-AGC-TCA-TCA-TCA-TCA-TCA-TCA-TCA-TCG-TCA-TCA-TCA-TCG-TCA-TCA-TCG-TCA-ACA-ACA-ACC-CCC-GCA-CCA-GCT-CCA-AAG-AAG-GCA-AGA-GAA-GCA-GAT-GGC-GGA-GAA-GAA-AAG-AAC-AAT-GAA-GAA-AGC-CAA-ACT-CCC-GCT-AGT-CCT-GGA-AGT-GGT-GGG-GTG-AGT-GAA-GGA-CAA-GAT-ACT-CAA-GGT-GGC-TCC-AAA-GGA-GAC-GCT-GAG-GAA-GGC-ACT-GAA-GAC-AAT-GAA-CAA-GCC-GAT-GAG-AGT-GCT-ACC-CAA-CCT-TCT-ACC-CCA-GGT-CAA-GGC-TCC-GTT-AAA-ACC-GAA-TCC-ACA-GAA-ACT-ACT-CCA-AAG-GAG-AAG-TGC-GGT-ACT-TCA-TTT-GTT-ATG-TGG-TTC-GGA-CAG-GGT-GTT-CCA-GTC-GCA-ACT-TTG-AAG-TGC-GGT-GAC-TAT-ACT-ATG-GTC-TAT-GCA-CCA-GAA-AAG-GAC-AAA-ACA-GAT-CCC-GCA-CCA-AGA-TAT-ATC-TCT-GGT-GAA-GTT-ACA-ACC-GTA-ACC-TTT-GAT-AAA-CAA-GAG-AGT-ACA-GTT-ACA-ATC-AAG-GTT-AAT-AAT-GTA-GAG-TTC-GGC-ACT-CTC-TCT-ACT-AGC-TCA-AGT-AAA-CCA-ACT-GAA-AAT-AAA-GGT-GAG-TCT-AGC-GAT-CAG-GTT-GGG-TCA-AGA-TCA-AGA-AGA-TCA-CTC-ACA-GAG-GAA-ACT-AGT-GAA-ACT-GCA-ACC-GTC-GAT-TTG-TTT-GCC-TTT-ACC-CTT-GAT-GGT-GGT-AAA-AGA-ATT-GAA-GTG-GCT-GTA-CCA-AGT-GAC-GAA-GAT-GTA-TCC-AAG-AGA-AAC-AAG-TAC-AGT-TTG-GTT-GCA-AAC-GAT-AAG-ACT-TTC-TAT-ACC-GGC-GCA-AAT-AGC-GGT-AAT-ACC-GAC-GGT-ATC-TAC-AGG-TTG-AAT-GAT-AAT-GGA-GAC-TTG-GTG-GAC-AAG-AAC-AAC-AAC-GTT-CTT-TTG-AAG-W-3′	MW480830	*C. hominis*	Ib
10	5′-YTT-TTT-TTT-TCA-CCC-AGC-CGT-TCC-ACT-CAG-AGG-CAC-CTT-GAA-AGA-TGT-TTC-TGT-TGA-GAG-CTC-ATC-ATC-ATC-ATC-ATC-ATC-ATC-GTC-ATC-ATC-ATC-GTC-ATC-ATC-GTC-AAC-AAC-AAC-CCC-CGC-ACC-AGC-TCC-AAA-GAA-GGC-AAG-AGA-AGC-AGA-TGG-CGG-AGA-AGA-AAA-GAA-CAA-TGA-AGA-AAG-CCA-AAC-TCC-CGC-TAG-TCC-TGG-AAG-TGG-TGG-GGT-GAG-TGA-AGG-ACA-AGA-TAC-TCA-AGG-TGG-CTC-CAA-AGG-AGA-CGC-TGA-GGA-AGG-CAC-TGA-AGA-CAA-TGA-ACA-AGC-CGA-TGA-GAG-TGC-TGC-CCA-ACC-TTC-TAC-CCC-AGG-TCA-AGG-CTC-CGT-TAA-AAC-CGA-ATC-CAC-AGA-AAC-TAC-TCC-AAA-GGA-GAA-GTG-CGG-TAC-TTC-ATT-TGT-TAT-GTG-GTT-CGG-ACA-GGG-TGT-TCC-AGT-CGC-AAC-TTT-GAA-GTG-CGG-TGA-CTA-TAC-TAT-GGT-CTA-TGC-ACC-AGA-AAA-GGA-CAA-AAC-AGA-TCC-CGC-ACC-AAG-ATA-TAT-CTC-TGG-TGA-AGT-TAC-AAC-CGT-AAC-CTT-TGA-TAA-ACA-AGA-GAG-TAC-AGT-TAC-AAT-CAA-GGT-TAA-TAA-TGT-AGA-GTT-CGG-CAC-TCT-CTC-TAC-TAG-CTC-AAG-TAA-ACC-AAC-TGA-AAA-TAA-AGG-TGA-GTC-TAG-CGA-TCA-GGT-TGG-GTC-AAG-ATC-AAG-AAG-ATC-ACT-CAC-AGA-GGA-AAC-TAG-TGA-AAC-TGC-AAC-CGT-CGA-TTT-GTT-TGC-CTT-TAC-CCT-TGA-TGG-TGG-TAA-AAG-AAT-TGA-AGT-GGC-TGT-ACC-AAG-TGA-CGA-AGA-TGT-ATC-CAA-GAG-AAA-CAA-GTA-CAG-TTT-GGT-TGC-AAA-CGA-TAA-GAC-TTT-CTA-TAC-CGG-CGC-AAA-TAG-CGG-TAA-TAC-CGA-CGG-TAT-CTA-CAG-GTT-GAA-TGA-TAA-TGG-AGA-CTT-GGT-GGA-CAA-GAA-CAA-CAA-CGT-TCT-TTT-GAA-GWG-KT-3′	MK105902	*C. hominis*	Ib
11	5′-TTC-TCA-GCC-CCA-GCC-GTT-CCA-CTC-AGA-GGC-ACC-TTG-AAA-GAT-GTT-TCT-GTT-GAG-AGC-TCA-TCA-TCA-TCA-TCA-TCA-TCA-TCG-TCA-TCA-TCA-TCG-TCA-TCA-TCG-TCA-ACA-ACA-ACC-CCC-GCA-CCA-GCT-CCA-AAG-AAG-GCA-AGA-GAA-GCA-GAT-GGC-GGA-GAA-GAA-AAG-AAC-AAT-GAA-GAA-AGC-CAA-ACT-CCC-GCT-AGT-CCT-GGA-AGT-GGT-GGG-GTG-AGT-GAA-GGA-CAA-GAT-ACT-CAA-GGT-GGC-TCC-AAA-GGA-GAC-GCT-GAG-GAA-GGC-ACT-GAA-GAC-AAT-GAA-CAA-GCC-GAT-GAG-AGT-GCT-ACC-CAA-CCT-TCT-ACC-CCA-GGT-CAA-GGC-TCC-GTT-AAA-ACC-GAA-TCC-ACA-GAA-ACT-ACT-CCA-AAG-GAG-AAG-TGC-GGT-ACT-TCA-TTT-GTT-ATG-TGG-TTC-GGA-CAG-GGT-GTT-CCA-GTC-GCA-ACT-TTG-AAG-TGC-GGT-GAC-TAT-ACT-ATG-GTC-TAT-GCA-CCA-GAA-AAG-GAC-AAA-ACA-GAT-CCC-GCA-CCA-AGA-TAT-ATC-TCT-GGT-GAA-GTT-ACA-ACC-GTA-ACC-TTT-GAT-AAA-CAA-GAG-AGT-ACA-GTT-ACA-ATC-AAG-GTT-AAT-AAT-GTA-GAG-TTC-GGC-ACT-CTC-TCT-ACT-AGC-TCA-AGT-AAA-CCA-ACT-GAA-AAT-AAA-GGT-GAG-TCT-AGC-GAT-CAG-GTT-GGG-TCA-AGA-TCA-AGA-AGA-TCA-CTC-ACA-GAG-GAA-ACT-AGT-GAA-ACT-GCA-ACC-GTC-GAT-TTG-TTT-GCC-TTT-ACC-CTT-GAT-GGT-GGT-AAA-AGA-ATT-GAA-GTG-GCT-GTA-CCA-AGT-GAC-GAAG-ATGT-ATC-CAA-GAG-AAA-CAA-GTA-CAG-TTT-GGT-TGC-AAA-CGA-TAA-GAC-TTT-CTA-TAC-CGG-CGC-AAA-TAG-CGG-TAA-TAC-CGA-CGG-TAT-CTA-CAG-GTT-GAA-TGA-TAA-TGG-AGA-CTT-GGT-GGA-CAA-GAA-CAA-CAA-CGT-TCT-TTT-GAA-GGA-TGT-GGG-3′	MT053132	*C. hominis*	Ib
12	5′-TWA-TTT-TCA-GCC-CCA-GCC-GTC-CCA-CTC-AGA-GGC-ACC-TTG-AAG-GAT-GTT-TCT-GTT-GAG-GGC-TCA-TCA-TCA-TCT-TCA-TCA-TCG-TCT-TCA-TCT-TCA-TCA-TCA-TCA-TCG-TCG-TCA-ACA-ACC-CCA-GCA-CCA-GCT-TCA-AAG-AAG-GTA-AGA-GAA-GCA-GAA-GGC-AGT-GAA-GAA-AAG-GAC-AGC-GAA-GAA-AAG-GAC-AGT-GAA-GAA-AAG-GGC-AGT-GAA-GAA-GGT-AGC-CAA-ACT-CCC-GCT-AGT-CCT-GGA-GGT-GGA-GGG-GTG-AGT-GAA-GGA-GAT-ACT-CAA-GGT-GAC-TCT-AAA-GGA-GAC-GGA-GTT-AGT-TCA-GAT-GAG-AAC-CAA-AGT-CAA-GGT-GGG-GAC-GCT-ACT-CCC-GGA-TCT-AGC-ACC-CAA-ACT-CAA-GCT-ACT-GAA-AAA-GAA-CCC-GGA-TCT-TCA-GAA-GCT-ACT-CCA-AAG-GAA-GAG-TGC-GGT-ACT-TCA-TTT-GTA-ATG-TGG-TTC-GGA-CAG-GGT-GTT-CCA-GTT-GTA-ACT-TTG-AAG-TGT-GGT-GGT-TAT-ACT-ATG-GTC-TAT-GCA-CCA-GAA-AAT-GGC-AAA-ACA-GAT-CCC-GCA-CCA-AGA-TAT-ATC-TCT-GGT-AAA-GTT-TCA-ACC-GTA-GAC-TTT-GAA-AAA-CAA-GAT-AGT-ACA-GTT-AAA-ATC-AAG-GTT-AAT-GGT-GTG-GAG-TTC-AGC-ACT-CTC-TCT-ACT-AGC-TCA-AGT-AAT-CCA-ACT-GAA-AAT-AGC-GGA-TCT-GAG-AGC-CAG-GCT-CAA-TCA-AGA-TCA-AGA-AGA-TCA-CTC-GCA-GAG-GAT-GGT-ACT-GAG-ACT-GCT-GCA-ACC-GTC-GAT-TTG-ATT-GCC-TTC-ACC-CTT-CAA-GGT-GGT-AAA-AGA-ATC-GAA-GTC-GCT-GTG-CCA-AGT-GAC-GAA-GAT-GTA-TCC-AAG-AGA-AAC-AAG-TAC-AGT-TTG-GTT-GCA-GGC-GAT-AAG-ACT-TTC-TAT-ACC-GGC-GCA-AAT-AGC-GGT-AAT-ACC-GAC-GGT-ATC-TAC-AGG-TTG-AAT-GAT-GAT-GGA-GAC-TTG-GTG-GAC-AAG-AAC-AAC-AAC-GTT-CTT-TTG-AAG-GAT-GTG-GTT-3′	GU214354	*C. hominis*	Ie
13	5′-CGC-TGT-WAT-TCT-CAG-CCC-CAG-CCG-TTC-CAC-TCA-GAG-GCA-CCT-TGA-AAG-ATG-TTT-CTG-TTG-AGA-GCT-CAT-CAT-CAT-CAT-CAT-CAT-CAT-CGT-CAT-CAT-CAT-CGT-CAT-CAT-CGT-CAA-CAA-CAA-CCC-CCG-CAC-CAG-CTC-CAA-AGA-AGG-CAA-GAG-AAG-CAG-ATG-GCG-GAG-AAG-AAA-AGA-ACA-ATG-AAG-AAA-GCC-AAA-CTC-CCG-CTA-GTC-CTG-GAA-GTG-GTG-GGG-TGA-GTG-AAG-GAC-AAG-ATA-CTC-AAG-GTG-GCT-CCA-AAG-GAG-ACG-CTG-AGG-AAG-GCA-CTG-AAG-ACA-ATG-AAC-AAG-CCG-ATG-AGA-GTG-CTA-CCC-AAC-CTT-CTA-CCC-CAG-GTC-AAG-GCT-CCG-TTA-AAA-CCG-AAT-CCA-CAG-AAA-CTA-CTC-CAA-AGG-AGA-AGT-GCG-GTA-CTT-CAT-TTG-TTA-TGT-GGT-TCG-GAC-AGG-GTG-TTC-CAG-TCG-CAA-CTT-TGA-AGT-GCG-GTG-ACT-ATA-CTA-TGG-TCT-ATG-CAC-CAG-AAA-AGG-ACA-AAA-CAG-ATC-CCG-CAC-CAA-GAT-ATA-TCT-CTG-GTG-AAG-TTA-CAA-CCG-TAA-CCT-TTG-ATA-AAC-AAG-AGA-GTA-CAG-TTA-CAA-TCA-AGG-TTA-ATA-ATG-TAG-AGT-TCG-GCA-CTC-TCT-CTA-CTA-GCT-CAA-GTA-AAC-CAA-CTG-AAA-ATA-AAG-GTG-AGT-CTA-GCG-ATC-AGG-TTG-GGT-CAA-GAT-CAA-GAA-GAT-CAC-TCA-CAG-AGG-AAA-CTA-GTG-AAA-CTG-CAA-CCG-TCG-ATT-TGT-TTG-CCT-TTA-CCC-TTG-ATG-GTG-GTA-AAA-GAA-TTG-AAG-TGG-CTG-TAC-CAA-GTG-ACG-AAG-ATG-TAT-CCA-AGA-GAA-ACA-AGT-ACA-GTT-TGG-TTG-CAA-ACG-ATA-AGA-CTT-TCT-ATA-CCG-GCG-CAA-ATA-GCG-GTA-ATA-CCG-ACG-GTA-TCT-ACA-GGT-TGA-ATG-ATA-ATG-GAG-ACT-TGG-TGG-ACA-AGA-ACA-ACA-ACG-TTC-TTT-TGA-AGG-ATG-CTG-TTT-YCC-TCT-GC-3′	MT053132	*C. hominis*	Ib
14	5′-TTT-TTC-AGC-CCC-AGC-CGT-TCC-ACT-CAG-AGG-CAC-CTT-GAA-AGA-TGT-TTC-TGT-TGA-GAG-CTC-ATC-ATC-ATC-ATC-ATC-ATC-ATC-GTC-ATC-ATC-ATC-GTC-ATC-ATC-GTC-AAC-AAC-AAC-CCC-CGC-ACC-AGC-TCC-AAA-GAA-GGC-AAG-AGA-AGC-AGA-TGG-CGG-AGA-AGA-AAA-GAA-CAA-TGA-AGA-AAG-CCA-AAC-TCC-CGC-TAG-TCC-TGG-AAG-TGG-TGG-GGT-GAG-TGA-AGG-ACA-AGA-TAC-TCA-AGG-TGG-CTC-CAA-AGG-AGA-CGC-TGA-GGA-AGG-CAC-TGA-AGA-CAA-TGA-ACA-AGC-CGA-TGA-GAG-TGC-TAC-CCA-ACC-TTC-TAC-CCC-AGG-TCA-AGG-CTC-CGT-TAA-AAC-CGA-ATC-CAC-AGA-AAC-TAC-TCC-AAA-GGA-GAA-GTG-CGG-TAC-TTC-ATT-TGT-TAT-GTG-GTT-CGG-ACA-GGG-TGT-TCC-AGT-CGC-AAC-TTT-GAA-GTG-CGG-TGA-CTA-TAC-TAT-GGT-CTA-TGC-ACC-AGA-AAA-GGA-CAA-AAC-AGA-TCC-CGC-ACC-AAG-ATA-TAT-CTC-TGG-TGA-AGT-TAC-AAC-CGT-AAC-CTT-TGA-TAA-ACA-AGA-GAG-TAC-AGT-TAC-AAT-CAA-GGT-TAA-TAA-TGT-AGA-GTT-CGG-CAC-TCT-CTC-TAC-TAG-CTC-AAG-TAA-ACC-AAC-TGA-AAA-TAA-AGG-TGA-GTC-TAG-CGA-TCA-GGT-TGG-GTC-AAG-ATC-AAG-AAG-ATC-ACT-CAC-AGA-GGA-AAC-TAG-TGA-AAC-TGC-AAC-CGT-CGA-TTT-GTT-TGC-CTT-TAC-CCT-TGA-TGG-TGG-TAA-AAG-AAT-TGA-AGT-GGC-TGT-ACC-AAG-TGA-CGA-AGA-TGT-ATC-CAA-GAG-AAA-CAA-GTA-CAG-TTT-GGT-TGC-AAA-CGA-TAA-GAC-TTT-CTA-TAC-CGG-CGC-AAA-TAG-CGG-TAA-TAC-CGA-CGG-TAT-CTA-CAG-GTT-GAA-TGA-TAA-TGG-AGA-CTT-GGT-GGA-CAA-GAA-CAA-CAA-CGT-TCT-TTT-GAA-GWW-G-3′	MT053132	*C. hominis*	Ib
15	5′-TTT-TTT-TYA-CCC-CAG-CCG-TTC-CAC-TCA-GAG-GCA-CCT-TGA-AAG-ATG-TTT-CTG-TTG-AGA-GCT-CAT-CAT-CAT-CAT-CAT-CAT-CAT-CGT-CAT-CAT-CAT-CGT-CAT-CAT-CGT-CAA-CAA-CAA-CCC-CCG-CAC-CAG-CTC-CAA-AGA-AGG-CAA-GAG-AAG-CAG-ATG-GCG-GAG-AAG-AAA-AGA-ACA-ATG-AAG-AAA-GCC-AAA-CTC-CCG-CTA-GTC-CTG-GAA-GTG-GTG-GGG-TGA-GTG-AAG-GAC-AAG-ATA-CTC-AAG-GTG-GCT-CCA-AAG-GAG-ACG-CTG-AGG-AAG-GCA-CTG-AAG-ACA-ATG-AAC-AAG-CCG-ATG-AGA-GTG-CTA-CCC-AAC-CTT-CTA-CCC-CAG-GTC-AAG-GCT-CCG-TTA-AAA-CCG-AAT-CCA-CAG-AAA-CTA-CTC-CAA-AGG-AGA-AGT-GCG-GTA-CTT-CAT-TTG-TTA-TGT-GGT-TCG-GAC-AGG-GTG-TTC-CAG-TCG-CAA-CTT-TGA-AGT-GCG-GTG-ACT-ATA-CTA-TGG-TCT-ATG-CAC-CAG-AAA-AGG-ACA-AAA-CAG-ATC-CCG-CAC-CAA-GAT-ATA-TCT-CTG-GTG-AAG-TTA-CAA-CCG-TAA-CCT-TTG-ATA-AAC-AAG-AGA-GTA-CAG-TTA-CAA-TCA-AGG-TTA-ATA-ATG-TAG-AGT-TCG-GCA-CTC-TCT-CTA-CTA-GCT-CAA-GTA-AAC-CAA-CTG-AAA-ATA-AAG-GTG-AGT-CTA-GCG-ATC-AGG-TTG-GGT-CAA-GAT-CAA-GAA-GAT-CAC-TCA-CAG-AGG-AAA-CTA-GTG-AAA-CTG-CAA-CCG-TCG-ATT-TGT-TTG-CCT-TTA-CCC-TTG-ATG-GTG-GTA-AAA-GAA-TTG-AAG-TGG-CTG-TAC-CAA-GTG-ACG-AAG-ATG-TAT-CCA-AGA-GAA-ACA-AGT-ACA-GTT-TGG-TTG-CAA-ACG-ATA-AGA-CTT-TCT-ATA-CCG-GCG-CAA-ATA-GCG-GTA-ATA-CCG-ACG-GTA-TCT-ACA-GGT-TGA-ATG-ATA-ATG-GAG-ACT-TGG-TGG-ACA-AGA-ACA-ACA-ACG-TTC-TTT-GAG-WGG-3′	MK105902	*C. hominis*	Ib
16	5′-CAG-CCC-CGG-CCG-TTS-CWC-TCA-GAG-GCA-CCT-YGA-AGG-ATG-TTT-CTG-TTG-AGG-GAT-CAT-CAT-CAT-CAT-CAT-CAT-CAT-CAT-CAT-CAT-CAT-CAT-CAT-CAT-CAT-CAA-CAT-CGA-CCG-TCG-CAC-CAG-CTC-CAA-AGA-AAG-AAA-GAA-CTG-TAG-AGG-GCG-GCA-CGG-AAG-GAA-AGA-ACG-AAG-AAA-GCA-GTC-CAG-GTT-CTG-AAG-AAC-AAG-ACG-GTG-GTA-AGG-AAG-ACG-GTG-GTA-AGG-AAA-ACG-GTG-AAG-GAG-ACA-CAG-TAG-ACG-GGG-AAC-AAA-CCG-GGA-GTG-GTT-CTC-AAG-TTA-CTC-CAT-CTG-GAA-GTG-CCG-GCA-CAG-CTA-CCG-AGT-CCA-CAG-CAA-CTA-CTA-CTC-CAA-AGG-AAG-AAT-GTG-GTA-CTT-CAT-TTG-TCA-TGT-GGT-TCG-AGA-AAG-GCA-CCC-CGG-TTG-CGA-CTT-TGA-AGT-GTG-GTG-ATT-ACA-CTA-TCG-TCT-ATG-CAC-CTA-TAA-AAG-ATC-AAA-CAG-ATC-CCG-CAC-CAA-GAT-ATA-TCT-CTG-GTG-AAG-TTA-CAT-CTG-TAT-CCT-TTG-AAA-AGA-GTG-AAA-GTA-CAG-TTA-CAA-TCA-AGG-TTA-ATG-GTA-AAG-AGT-TCA-GCA-CTC-TCT-CTG-CTA-ACT-CAA-GTA-GTC-CAA-CTA-AAG-ATA-ACG-GTG-AAT-CTA-GTG-ACA-GTC-AGG-TTC-AAT-CAA-GAT-CAA-GAA-GAT-CAC-TCG-CAG-AGG-AGA-ATG-GTG-AAA-CAG-TTG-CAA-CAG-TTG-ATT-TGT-TTG-CCT-TTA-CTC-TTG-ATG-GTG-GTA-GAA-GAA-TTG-AAG-TGG-CTG-TGC-CAA-AGG-ACG-AAA-ATG-CAG-ACA-AAA-GAA-GTG-AGT-ACA-GTT-TGG-TTG-CAG-ACG-ATA-AGC-CTT-TCT-ATA-CCG-GCG-CAA-ACA-GTG-GCA-TCA-CCA-ATG-GTG-TCT-ACA-AAT-TGG-ATG-AGA-ATG-GAA-ACT-TGG-WTG-ACR-AGG-ACA-ACA-AAG-TTC-TCT-TGA-AAG-ATG-3′	KY990897	*C. hominis*	Ia
17	5′-TAC-CGC-TAT-TTG-CGC-CGG-TAT-AGA-AAG-TCT-TAT-CGT-TTG-CAA-CCA-ARC-WGT-ACT-TGT-TTC-TCT-GGA-KAC-ATC-TTC-GTC-ACW-TGG-TAC-AGC-CAY-TTC-AAT-TCT-TTT-WCC-ACC-ATC-AAG-GGT-AAA-GGC-AAR-CAA-ATM-GAC-GGT-TGC-AGT-TTC-AST-AGT-TTC-CTC-TGT-GAG-TGA-TCT-TCT-TGA-TCW-TGA-CCC-AAC-CTG-ATC-GCT-AGA-CTC-ACC-TTT-ATT-TTC-AGT-TGG-TKT-ACW-TGA-GCT-AGT-AGA-GAG-AGT-GCC-GAM-CTC-TAC-ATT-ATT-AAC-CTT-GAT-KGT-ARC-TGT-ACT-CTC-TTG-TTW-ATC-AAA-GGT-TAC-GGT-TGK-AAC-3′	OP778252	*C. hominis*	Ib
18	5′-ACA-ACC-CCC-ACA-CCA-GCT-CCA-AAG-AAG-GCA-AGA-GAA-GCA-GAT-GGC-GGA-GAA-GAA-AAG-ATC-AAC-GAA-GAA-AGC-CAA-ACT-CCC-GCT-AGT-CCT-GGA-AGT-GGT-GGG-GTG-AGT-GAA-GGA-CAA-GAT-ACT-CAA-GGT-GGC-TCC-AAA-GGA-GAC-GCT-GAG-GAA-GGC-ACT-GAA-GTC-AAT-GAA-CAA-CCC-GAT-GAG-AGT-GCT-ACC-CAA-CCT-TCT-ACC-CCA-GGT-CAA-GGC-TCC-GTT-AAA-ACC-GAA-TCC-ACA-GAA-ACT-ACT-CCA-AAG-GAG-AAG-TGC-GGT-ACT-TCA-TTT-GTT-ATG-TGG-TTC-GGA-CAG-GGT-GTT-CCA-GTC-GCA-ACT-TTG-AAG-TGC-GGT-GAC-TAT-ACT-ATG-GTC-TAT-GCA-CCA-GAA-AAG-GAC-AAA-ACA-GAT-CCC-GCA-CCA-AGA-TAT-ATC-TCT-GGT-GAA-GTT-ACA-ACC-GTA-ACY-TTC-VAA-ARV-MAC-SAC-MMY-MCA-RDC-CCC-CCC-GCM-WCT-ATA-GTC-TCC-GCT-GTA-TTY-TTT-GTA-ACG-CTG-AAT-GAA-TCG-ATC-AGC-TCA-CTG-TMT-GGT-GCA-TCG-CTC-TCT-YCC-GAA-AGA-TTA-TAC-ACA-TTT-ACA-GAA-ATC-TTT-TGA-TCT-GTC-ACA-TCA-AAA-ATA-GCA-TAA-CTT-GGA-CTA-TAC-TCC-TGA-TTC-CAC-TCT-TGA-TTT-CCC-ACA-GGA-AGA-KTT-CCT-TCC-AGA-TAG-GAT-GCA-GAA-CCA-CTC-TCT-CCA-TTT-GCT-AAA-ATC-KTT-SC-3′	OP778252	*C. hominis*	Ib
19	5′-KKT-TTT-TTC-AGC-CCC-AGC-CGT-TCC-ACT-CAG-AGG-CAC-CTT-GAA-AGA-TGT-TTC-TGT-TGA-GAG-CTC-ATC-ATC-ATC-ATC-ATC-ATC-ATC-GTC-ATC-ATC-ATC-GTC-ATC-ATC-GTC-AAC-AAC-AAC-CCC-CGC-ACC-AGC-TCC-AAA-GAA-GGC-AAG-AGA-AGC-AGA-TGG-CGG-AGA-AGA-AAA-GAA-CAA-TGA-AGA-AAG-CCA-AAC-TCC-CGC-TAG-TCC-TGG-AAG-TGG-TGG-GGT-GAG-TGA-AGG-ACA-AGA-TAC-TCA-AGG-TGG-CTC-CAA-AGG-AGA-CGC-TGA-GGA-AGG-CAC-TGA-AGA-CAA-TGA-ACA-AGC-CGA-TGA-GAG-TGC-TAC-CCA-ACC-TTC-TAC-CCC-AGG-TCA-AGG-CTC-CGT-TAA-AAC-CGA-ATC-CAC-AGA-AAC-TAC-TCC-AAA-GGA-GAA-GTG-CGG-TAC-TTC-ATT-TGT-TAT-GTG-GTT-CGG-ACA-GGG-TGT-TCC-AGT-CGC-AAC-TTT-GAA-GTG-CGG-TGA-CTA-TAC-TAT-GGT-CTA-TGC-ACC-AGA-AAA-GGA-CAA-AAC-AGA-TCC-CGC-ACC-AAG-ATA-TAT-CTC-TGG-TGA-AGT-TAC-AAC-CGT-AAC-CTT-TGA-TAA-ACA-AGA-GAG-TAC-AGT-TAC-AAT-CAA-GGT-TAA-TAA-TGT-AGA-GTT-CGG-CAC-TCT-CTC-TAC-TAG-CTC-AAG-TAA-ACC-AAC-TGA-AAA-TAA-AGG-TGA-GTC-TAG-CGA-TCA-GGT-TGG-GTC-AAG-ATC-AAG-AAG-ATC-ACT-CAC-AGA-GGA-AAC-TAG-TGA-AAC-TGC-AAC-CGT-CGA-TTT-GTT-TGC-CTT-TAC-CCT-TGA-TGG-TGG-TAA-AAG-AAT-TGA-AGT-GGC-TGT-ACC-AAG-TGA-CGA-AGA-TGT-ATC-CAA-GAG-AAA-CAA-GTA-CAG-TTT-GGT-TGC-AAA-CGA-TAA-GAC-TTT-CTA-TAC-CGG-CGC-AAA-TAG-CGG-TAA-TAC-CGA-CGG-TAT-CTA-CAG-GTT-GAA-TGA-TAA-TGG-AGA-CTT-GGT-GGA-CAA-GAAC-AAC-AAC-GTT-CTT-TTG-AAG-GAT-GTG-GGK-3′	MT053132	*C. hominis*	Ib
20	5′-TTT-TTT-TTY-ACC-CAG-CCG-TTC-CAC-TCA-GAG-GCA-CCT-TGA-AAG-ATG-TTT-CTG-TTG-AGA-GCT-CAT-CAT-CAT-CAT-CAT-CAT-CAT-CGT-CAT-CAT-CAT-CGT-CAT-CAT-CGT-CAA-CAA-CAA-CCC-CCG-CAC-CAG-CTC-CAA-AGA-AGG-CAA-GAG-AAG-CAG-ATG-GCG-GAG-AAG-AAA-AGA-ACA-ATG-AAG-AAA-GCC-AAA-CTC-CCG-CTA-GTC-CTG-GAA-GTG-GTG-GGG-TGA-GTG-AAG-GAC-AAG-ATA-CTC-AAG-GTG-GCT-CCA-AAG-GAG-ACG-CTG-AGG-AAG-GCA-CTG-AAG-ACA-ATG-AAC-AAG-CCG-ATG-AGA-GTG-CTA-CCC-AAC-CTT-CTA-CCC-CAG-GTC-AAG-GCT-CCG-TTA-AAA-CCG-AAT-CCA-CAG-AAA-CTA-CTC-CAA-AGG-AGA-AGT-GCG-GTA-CTT-CAT-TTG-TTA-TGT-GGT-TCG-GAC-AGG-GTG-TTC-CAG-TCG-CAA-CTT-TGA-AGT-GCG-GTG-ACT-ATA-CTA-TGG-TCT-ATG-CAC-CAG-AAA-AGG-ACA-AAA-CAG-ATC-CCG-CAC-CAA-GAT-ATA-TCT-CTG-GTG-AAG-TTA-CAA-CCG-TAA-CCT-TTG-ATA-AAC-AAG-AGA-GTA-CAG-TTA-CAA-TCA-AGG-TTA-ATA-ATG-TAG-AGT-TCG-GCA-CTC-TCT-CTA-CTA-GCT-CAA-GTA-AAC-CAA-CTG-AAA-ATA-AAG-GTG-AGT-CTA-GCG-ATC-AGG-TTG-GGT-CAA-GAT-CAA-GAA-GAT-CAC-TCA-CAG-AGG-AAA-CTA-GTG-AAA-CTG-CAA-CCG-TCG-ATT-TGT-TTG-CCT-TTA-CCC-TTG-ATG-GTG-GTA-AAA-GAA-TTG-AAG-TGG-CTG-TAC-CAA-GTG-ACG-AAG-ATG-TAT-CCA-AGA-GAA-ACA-AGT-ACA-GTT-TGG-TTG-CAA-ACG-ATA-AGA-CTT-TCT-ATA-CCG-GCG-CAA-ATA-GCG-GTA-ATA-CCG-ACG-GTA-TCT-ACA-GGT-TGA-ATG-ATA-ATG-GAG-ACT-TGG-TGG-ACA-AGA-ACA-ACA-ACG-TTC-TTT-GAG-RGG-K-3′	MK105902	*C. hominis*	Ib
21	5′-ACC-CAG-CCG-TCC-CAC-TCA-GAG-GCA-CCT-TGA-AGG-ATG-TTT-CTG-TTG-AGG-GCT-CAT-CAT-CAT-CTT-CAT-CAT-CGT-CTT-CAT-CTT-CAT-CAT-CAT-CAT-CGT-CGT-CAA-CAA-CCC-CAG-CAC-CAG-CTT-CAA-AGA-AGG-TAA-GAG-AAG-CAG-AAG-GCA-GTG-AAG-AAA-AGG-ACA-GCG-AAG-AAA-AGG-ACA-GTG-AAG-AAA-AGG-GCA-GTG-AAG-AAG-GTA-GCC-AAA-CTC-CCG-CTA-GTC-CTG-GAG-GTG-GAG-GGG-TGA-GTG-AAG-GAG-ATA-CTC-AAG-GTG-ACT-CTA-AAG-GAG-ACG-GAG-TTA-GTT-CAG-ATG-AGA-ACC-AAA-GTC-AAG-GTG-GGG-ACG-CTA-CTC-CCG-GAT-CTA-GCA-CCC-AAA-CTC-AAG-CTA-CTG-AAA-AAG-AAC-CCG-GAT-CTT-CAG-AAG-CTA-CTC-CAA-AGG-AAG-AGT-GCG-GTA-CTT-CAT-TTG-TAA-TGT-GGT-TCG-GAC-AGG-GTG-TTC-CAG-TTG-TAA-CTT-TGA-AGT-GTG-GTG-GTT-ATA-CTA-TGG-TCT-ATG-CAC-CAG-AAA-ATG-GCA-AAA-CAG-ATC-CCG-CAC-CAA-GAT-ATA-TCT-CTG-GTA-AAG-TTT-CAA-CCG-TAG-ACT-TTG-AAA-AAC-AAG-ATA-GTA-CAG-TTA-AAA-TCA-AGG-TTA-ATG-GTG-TGG-AGT-TCA-GCA-CTC-TCT-CTA-CTA-GCT-CAA-GTA-ATC-CAA-CTG-AAA-ATA-GCG-GAT-CTG-AGA-GCC-AGG-CTC-AAT-CAA-GAT-CAA-GAA-GAT-CAC-TCG-CAG-AGG-ATG-GTA-CTG-AGA-CTG-CTG-CAA-CCG-TCG-ATT-TGA-TTG-CCT-TCA-CCC-TTC-AAG-GTG-GTA-AAA-GAA-TCG-AAG-TCG-CTG-TGC-CAA-GTG-ACG-AAG-ATG-TAT-CCA-AGA-GAA-ACA-AGT-ACA-GTT-TGG-TTG-CAG-GCG-ATA-AGA-CTT-TCT-ATA-CCG-GCG-CAA-ATA-GCG-GTA-ATA-CCG-ACG-GTA-TCT-ACA-GGT-TGA-ATG-ATG-ATG-GAG-ACT-TGG-TGG-ACA-AGA-ACA-ACA-ACG-TTC-TTT-GAA-GWS-KKK-3′	ON863660	*C. hominis*	Ie
22	5′-CTT-TTT-TTY-ACC-CAG-CCG-TTC-CAC-TCA-GAG-GCA-CTT-TAA-AGG-ATG-TTT-CTG-TTG-AGA-GCT-CAT-CGT-CAT-CAT-CGT-CAT-CGT-CAA-CAA-CAA-CCC-CCG-CAC-CAG-CTC-CAA-AGA-AGG-TAA-GAG-AAA-GCG-AAG-AAG-GGA-AGA-ACA-GTG-AAG-ATA-GTC-AGA-CTC-CCG-CTA-GTC-CTG-GAA-GTG-ATT-CTC-AGG-ATA-GCT-CTA-AAG-GAG-ACG-AAG-CTG-TAG-ATG-GAT-CCG-CTT-CCG-GAT-CTA-GTA-CCC-CAA-CTC-AAG-CTG-CTG-AAA-AGG-AGC-CCG-AAA-CTC-CAG-AAT-CTA-CTC-CAA-AGG-AAG-AAT-GTG-GTA-CTT-CAT-TTA-TAA-TGT-GGT-TCG-GAG-AAG-GTA-CTC-CAG-CCA-CAA-CTT-TGA-AGT-GCG-GTG-GCT-ACA-CTA-TCG-TCT-ATG-CAC-CAG-AAA-AGG-ATA-ATA-AAG-AAC-CCG-CAC-CAA-GAT-ACA-TCT-CTG-GTG-ATG-TTA-AGG-CTG-TAA-CCT-TTG-AAA-AGG-GAG-AAG-ATA-ATA-CAG-TTA-AAA-TCA-AGG-TTG-ATG-GTA-AGG-AGT-TCA-GTA-CTC-TCT-CTT-CTA-GCT-CAA-GCA-GTC-CAA-CTG-AAA-ATA-ACG-GAT-CTA-CGG-GCC-AGG-TTG-CAT-CAA-GAT-CAA-GAA-GAT-CGC-TCT-CAG-AGG-AAA-ATA-GTG-AAA-CTG-CTG-CAA-CCG-TCG-ATT-TGT-TTG-CCT-TCA-CCC-TTG-ATG-GTG-GCC-GAA-GAA-TTG-AAG-TTG-CTG-TAC-CCA-GCG-TCG-AAG-ATG-CAA-CCA-AAA-GAG-ACA-AGT-ACA-GTT-TGG-TTG-CAA-ACG-GTA-AGC-CTT-TCT-ATA-CCG-GCG-CAA-ACA-GCG-GCA-CTA-CCA-ATG-GTG-TCT-ACA-GGT-TGA-ATG-AGA-ACG-GAG-ACT-TGG-TTG-ACA-AGG-ACA-ACA-CAG-TTC-TCT-TGA-AGA-GKG-3′	KU670811	*C. parvum*	IIc
23	5′-TCY-CTC-AGA-GCA-CCT-TGA-AGG-ATG-TTT-CTG-TTG-AGG-RCT-CAT-CAT-CAT-CAT-CAT-CAT-CAT-CAT-CAT-CAT-CAT-CAT-CAT-CAT-CAA-CGA-CCG-TCG-CAC-CAG-CTT-CAA-ATA-AGG-CAA-GAA-CTG-GAG-AGG-ACA-CAG-GAC-GAA-GCG-AAG-GAA-GTC-AAG-GTT-CTG-AAG-AAC-ACC-AAG-ACG-GAG-AGG-ACG-ATA-GTT-CAG-ATT-CTA-GTG-GAG-GCA-GTG-TAG-GAG-GCA-CAG-AGA-GCG-GAA-GTG-CAG-GAG-GAA-AGA-ACG-AAG-AAG-ATA-GTT-CAA-GTT-CTG-GAG-GTG-CTC-AGG-ACG-GCA-GTG-GAG-GCA-CTG-CAG-AAG-GCG-CTA-CTC-AGT-CCG-AGG-CTA-CTG-CTT-CTC-AAG-GTG-CTC-CAT-CTC-AAG-GTT-CTG-ACA-AAA-CTA-CCG-AGT-CCA-CAC-AAA-CTA-CTC-CAA-AGG-AAG-AGT-GCG-GTA-CTT-CGT-TTG-TAA-TGT-GGT-TCG-GTG-AAG-GTA-CCC-CGG-TTG-CGA-CCT-TGA-AGT-GTG-GTG-GTT-ACA-CTA-TCG-TCT-ATG-CAC-CTG-TAA-AGG-ATC-AAG-CAA-ATC-CCG-CAC-CAA-GAT-ATA-TCT-CTG-GTG-AAG-TAA-AGA-ATG-TAT-CCT-TCC-AAA-AAG-AAA-GTG-ATA-ATA-CAA-TTA-AAA-TCA-AGG-TTG-ACG-GTC-AGG-ATT-TCA-GCA-CTC-TCT-CTG-CTA-GCT-CAA-GTA-GTC-CAA-CCG-AAA-ATA-AAG-GTG-AGT-CTG-GCA-ATC-AGG-TTG-AGT-CAA-GAT-CAA-GAA-GAT-CAC-TCA-CAG-AGG-AAA-CTA-GTG-AAA-CTG-CAA-CCG-TCG-ATT-TGT-TTG-CCT-TTA-CCC-TTA-ATG-GTG-GTA-AGA-GAA-TTG-AAG-TGG-CTG-TGC-CAA-ACG-CCG-AAG-AAA-CAT-CGA-AAA-GAG-ACA-AGT-ACA-GTT-TGG-TTG-CAG-ACG-ATA-GTG-CTT-TCT-ATA-CCG-GCA-AAA-ATA-GCG-GCA-GCA-CCG-ATG-GTG-TCT-ACA-AGT-TGA-ATG-AGA-ACG-GAG-ACT-TAG-T-3′	MK982538	*C. hominis*	Id
24	5′-KTT-TTT-CTC-AGC-CCC-AGC-CGT-TCC-ACT-CAG-AGG-CAC-CTT-GAA-AGA-TGT-TTC-TGT-TGA-GAG-CTC-ATC-ATC-ATC-ATC-ATC-ATC-ATC-GTC-ATC-ATC-ATC-GTC-ATC-ATC-GTC-AAC-AAC-AAC-CCC-CGC-ACC-AGC-TCC-AAA-GAA-GGC-AAG-AGA-AGC-AGA-TGG-CGG-AGA-AGA-AAA-GAA-CAA-TGA-AGA-AAG-CCA-AAC-TCC-CGC-TAG-TCC-TGG-AAG-TGG-TGG-GGT-GAG-TGA-AGG-ACA-AGA-TAC-TCA-AGG-TGG-CTC-CAA-AGG-AGA-CGC-TGA-GGA-AGG-CAC-TGA-AGA-CAA-TGA-ACA-AGC-CGA-TGA-GAG-TGC-TAC-CCA-ACC-TTC-TAC-CCC-AGG-TCA-AGG-CTC-CGT-TAA-AAC-CGA-ATC-CAC-AGA-AAC-TAC-TCC-AAA-GGA-GAA-GTG-CGG-TAC-TTC-ATT-TGT-TAT-GTG-GTT-CGG-ACA-GGG-TGT-TCC-AGT-CGC-AAC-TTT-GAA-GTG-CGG-TGA-CTA-TAC-TAT-GGT-CTA-TGC-ACC-AGA-AAA-GGA-CAA-AAC-AGA-TCC-CGC-ACC-AAG-ATA-TAT-CTC-TGG-TGA-AGT-TAC-AAC-CGT-AAC-CTT-TGA-TAA-ACA-AGA-GAG-TAC-AGT-TAC-AAT-CAA-GGT-TAA-TAA-TGT-AGA-GTT-CGG-CAC-TCT-CTC-TAC-TAG-CTC-AAG-TAA-ACC-AAC-TGA-AAA-TAA-AGG-TGA-GTC-TAG-CGA-TCA-GGT-TGG-GTC-AAG-ATC-AAG-AAG-ATC-ACT-CAC-AGA-GGA-AAC-TAG-TGA-AAC-TGC-AAC-CGT-CGA-TTT-GTT-TGC-CTT-TAC-CCT-TGA-TGG-TGG-TAA-AAG-AAT-TGA-AGT-GGC-TGT-ACC-AAG-TGA-CGA-AGA-TGT-ATC-CAA-GAG-AAA-CAA-GTA-CAG-TTT-GGT-TGC-AAA-CGA-TAA-GAC-TTT-CTA-TAC-CGG-CGC-AAA-TAG-CGG-TAA-TAC-CGA-CGG-TAT-CTA-CAG-GTT-GAA-TGA-TAA-TGG-AGA-CTT-GGT-GGA-CAA-GAA-CAA-CAA-CGT-TCT-TTT-GAA-GGW-GTG-G-3′	MT053132	*C. hominis*	Ib
25	5′-TTT-TTT-MYA-CCC-CGG-CCG-TTC-CAC-TCA-GAG-GCA-CCT-TGA-AGG-ATG-TTT-CTG-TTG-AGG-GAT-CAT-CGT-CAT-CGT-CAT-CAT-CAT-CAT-CAT-CAT-CAT-CAT-CAT-CAT-CAT-CAA-CAT-CGA-CCG-TCG-CAC-CAG-CTC-CAA-AGA-AAG-AAA-GAA-CTG-TAG-AGG-GCA-CGG-AAG-GAA-AGA-ACG-AAG-AAA-GCA-GTC-CAG-GTT-CTG-AAG-AAC-AAG-ACG-GTG-GTA-AGG-AAG-ACG-GTG-GTA-AGG-AAG-ACG-GTG-GTA-AGG-AAG-ACG-GTG-GTA-AGG-AAG-ACG-CTG-AAG-AAG-GAG-ACA-CAG-TAG-ACG-GGG-AAC-AAA-CCG-GGA-GTG-GTT-CTC-AAG-TTA-CTC-CAT-CTG-AAA-GTG-CCG-GCA-CAG-CTA-CCG-AGT-CCA-CAG-CAA-CTA-CTA-CTC-CAA-AGG-AAG-AAT-GTG-GTA-CTT-CAT-TTG-TCA-TGT-GGT-TCG-AGC-AAG-GCA-CCC-CGG-TTG-CGA-CCT-TGA-AGT-GTG-GTG-ATT-ACA-CTA-TCG-TCT-ATG-CAC-CTA-TAA-AAG-ATC-AAA-CAG-ATC-CCG-CAC-CAA-GAT-ATA-TCT-CTG-GTG-AAG-TTA-CAT-CTG-TAT-CCT-TTG-AAA-AGA-GTG-AAA-GTA-CAG-TTA-CAA-TCA-AGG-TTA-ATG-GTA-AAG-AGT-TCA-GCA-CTC-TCT-CTG-CTA-ACT-CAA-GTA-GTC-CAA-CTA-AAG-ATA-ACG-GTG-AAT-CTA-GTG-ACA-GTC-GGG-TTC-AAT-CVA-GAT-CAA-GAA-GAT-CAC-TCG-CAG-AGG-AGA-ATG-GTG-AAA-CAG-YTG-CAA-CCG-TTG-ATT-TGT-TTG-CCT-TTA-CTC-TTG-ATG-GTG-GTA-AAA-KAA-TTG-ARG-TGG-CTG-TGC-CAR-AGG-ACG-AAA-ATG-MMG-ACA-AAA-GAA-GCG-AGT-ACA-GTT-TGG-TTG-CAG-ACG-ATA-AGC-CTT-TCT-ATA-CCG-GCG-CAA-ACA-GTG-GCA-CCA-CCR-ATG-GTG-TCT-ACA-AAT-TGG-ATG-AKA-ATG-GAA-ACT-TGG-TTG-ACR-AGG-ACA-ACA-AAG-TTC-TCT-TGA-AAT-GTG-K-3′	MN661180	*C. hominis*	Ia
26	5′-KKT-TTT-TTC-AGC-CCC-AGC-CGT-TCC-ACT-CAG-AGG-CAC-CTT-GAA-AGA-TGT-TTC-TGT-TGA-GAG-CTC-ATC-ATC-ATC-ATC-ATC-ATC-ATC-GTC-ATC-ATC-ATC-GTC-ATC-ATC-GTC-AAC-AAC-AAC-CCC-CGC-ACC-AGC-TCC-AAA-GAA-GGC-AAG-AGA-AGC-AGA-TGG-CGG-AGA-AGA-AAA-GAA-CAA-TGA-AGA-AAG-CCA-AAC-TCC-CGC-TAG-TCC-TGG-AAG-TGG-TGG-GGT-GAG-TGA-AGG-ACA-AGA-TAC-TCA-AGG-TGG-CTC-CAA-AGG-AGA-CGC-TGA-GGA-AGG-CAC-TGA-AGA-CAA-TGA-ACA-AGC-CGA-TGA-GAG-TGC-TAC-CCA-ACC-TTC-TAC-CCC-AGG-TCA-AGG-CTC-CGT-TAA-AAC-CGA-ATC-CAC-AGA-AAC-TAC-TCC-AAA-GGA-GAA-GTG-CGG-TAC-TTC-ATT-TGT-TAT-GTG-GTT-CGG-ACA-GGG-TGT-TCC-AGT-CGC-AAC-TTT-GAA-GTG-CGG-TGA-CTA-TAC-TAT-GGT-CTA-TGC-ACC-AGA-AAA-GGA-CAA-AAC-AGA-TCC-CGC-ACC-AAG-ATA-TAT-CTC-TGG-TGA-AGT-TAC-AAC-CGT-AAC-CTT-TGA-TAA-ACA-AGA-GAG-TAC-AGT-TAC-AAT-CAA-GGT-TAA-TAA-TGT-AGA-GTT-CGG-CAC-TCT-CTC-TAC-TAG-CTC-AAG-TAA-ACC-AAC-TGA-AAA-TAA-AGG-TGA-GTC-TAG-CGA-TCA-GGT-TGG-GTC-AAG-ATC-AAG-AAG-ATC-ACT-CAC-AGA-GGA-AAC-TAG-TGA-AAC-TGC-AAC-CGT-CGA-TTT-GTT-TGC-CTT-TAC-CCT-TGA-TGG-TGG-TAA-AAG-AAT-TGA-AGT-GGC-TGT-ACC-AAG-TGA-CGA-AGA-TGT-ATC-CAA-GAG-AAA-CAA-GTA-CAG-TTT-GGT-TGC-AAA-CGA-TAA-GAC-TTT-CTA-TAC-CGG-CGC-AAA-TAG-CGG-TAA-TAC-CGA-CGG-TAT-CTA-CAG-GTT-GAA-TGA-TAA-TGG-AGA-CTT-GGT-GGA-CAA-GAA-CAA-CAA-CGT-TCT-TTT-GAA-GRW-GT-3′	MT053132	*C. hominis*	Ib
27	5′-TTT-TTC-AGC-CCC-AGC-CGT-TCC-ACT-CAG-AGG-CAC-CTT-GAA-AGA-TGT-TTC-TGT-TGA-GAG-CTC-ATC-ATC-ATC-ATC-ATC-ATC-ATC-GTC-ATC-ATC-ATC-GTC-ATC-ATC-GTC-AAC-AAC-AAC-CCC-CGC-ACC-AGC-TCC-AAA-GAA-GGC-AAG-AGA-AGC-AGA-TGG-CGG-AGA-AGA-AAA-GAA-CAA-TGA-AGA-AAG-CCA-AAC-TCC-CGC-TAG-TCC-TGG-AAG-TGG-TGG-GGT-GAG-TGA-AGG-ACA-AGA-TAC-TCA-AGG-TGG-CTC-CAA-AGG-AGA-CGC-TGA-GGA-AGG-CAC-TGA-AGA-CAA-TGA-ACA-AGC-CGA-TGA-GAG-TGC-TAC-CCA-ACC-TTC-TAC-CCC-AGG-TCA-AGG-CTC-CGT-TAA-AAC-CGA-ATC-CAC-AGA-AAC-TAC-TCC-AAA-GGA-GAA-GTG-CGG-TAC-TTC-ATT-TGT-TAT-GTG-GTT-CGG-ACA-GGG-TGT-TCC-AGT-CGC-AAC-TTT-GAA-GTG-CGG-TGA-CTA-TAC-TAT-GGT-CTA-TGC-ACC-AGA-AAA-GGA-CAA-AAC-AGA-TCC-CGC-ACC-AAG-ATA-TAT-CTC-TGG-TGA-AGT-TAC-AAC-CGT-AAC-CTT-TGA-TAA-ACA-AGA-GAG-TAC-AGT-TAC-AAT-CAA-GGT-TAA-TAA-TGT-AGA-GTT-CGG-CAC-TCT-CTC-TAC-TAG-CTC-AAG-TAA-ACC-AAC-TGA-AAA-TAA-AGG-TGA-GTC-TAG-CGA-TCA-GGT-TGG-GTC-AAG-ATC-AAG-AAG-ATC-ACT-CAC-AGA-GGA-AAC-TAG-TGA-AAC-TGC-AAC-CGT-CGA-TTT-GTT-TGC-CTT-TAC-CCT-TGA-TGG-TGG-TAA-AAG-AAT-TGA-AGT-GGC-TGT-ACC-AAG-TGA-CGA-AGA-TGT-ATC-CAA-GAG-AAA-CAA-GTA-CAG-TTT-GGT-TGC-AAA-CGA-TAA-GAC-TTT-CTA-TAC-CGG-CGC-AAA-TAG-CGG-TAA-TAC-CGA-CGG-TAT-CTA-CAG-GTT-GAA-TGA-TAA-TGG-AGA-CTT-GGT-GGA-CAA-GAA-CAA-CAA-CGT-TCT-TTT-GAA-GWG-GGG-3′	MT053132	*C. hominis*	Ib
28	5′-TYT-GGT-GAG-GTA-AAA-AAT-GTA-TCC-TTC-CAA-AAA-GAA-AGY-GAT-GGT-ACA-WTT-AAA-ATC-AAG-ATT-GAS-GAA-AAG-GAG-TTC-AGT-TCT-CTY-TAR-YYG-AST-CAA-GCA-CTC-CRA-MYK-CAW-ATA-GYG-GAT-YCS-CGG-AAC-AGG-TKC-AWT-CAA-GAK-CAA-GAA-GAT-CAC-TCA-CAG-AGG-GAA-GTG-AAA-CWC-SKG-CTS-CCG-TMG-ATT-TGT-YYG-CCT-TCA-CCC-TTG-ATG-GTG-GTA-AAA-GAA-TTG-AAG-TGA-MTT-TCC-GCT-GTA-TTC-TCA-GCC-AGT-ATC-TCG-TAC-CCT-GCC-CTG-CCG-TTG-TAT-TCC-TGA-AGC-CAG-AAA-TCT-TCA-CTG-TAA-GCA-GTT-CCG-TGC-TCC-TGC-TGG-ATT-TGA-ATC-TTT-GTG-TCA-TAC-TCG-ACC-GAC-TTC-ATG-CAG-TCA-AGA-TAT-TCT-TCT-CTG-CTG-ATG-CTG-GTT-CCT-CTG-CAA-SGY-3′	JF268649	*C. parvum*	IIe
29	5′-TTT-TTT-YAC-CCA-GCC-GTT-CCA-CTC-AGA-GGC-ACT-TTA-AAG-GAT-GCT-TCT-GTT-GAG-GGC-TCA-TCA-TCA-TCA-TCA-TCA-TCA-TCA-TCA-TCA-TCG-ACC-ACC-GTC-GCA-CCA-GCT-CCA-AAG-AAA-GAA-AGA-ACT-GGA-GAG-GGC-GTA-GAT-GGA-AAG-GAC-CAA-GTA-GAT-AGT-ACA-GGT-TCT-GAT-CAG-AAC-AGT-AAA-GGA-GAC-ACT-AAA-GGA-ACC-ACA-GAA-GAT-GGT-AAA-GAG-ACC-GAA-GGT-ACT-GTT-TCC-AAA-CCC-ACT-ACT-CCA-GAT-CAA-GGT-GAG-AGC-GCA-ACT-CCC-GGA-TCC-ACG-GAA-ACT-ACT-CCA-AAG-GAA-GAA-TGC-GGT-ACT-TCA-TTT-GTA-ATG-TGG-TTC-GGA-GAA-GGT-ACC-CCA-GTT-GCG-ACC-TTG-AAG-TGT-GGT-GGT-TAC-ACT-ATC-GTC-TAT-GCA-CCT-GTA-AAG-GAA-CAA-ACA-AAT-CCC-GCA-CCA-AGA-TAT-ATC-TCT-GGT-GAG-GTA-AAA-AAT-GTA-TCC-TTC-CAA-AAA-GAA-AGT-GAT-GGT-ACA-ATT-AAA-ATC-AAG-ATT-GAC-GAA-AAG-GAG-TTC-AGT-TCT-CTC-TCT-ACT-GAC-TCA-AGC-ACT-CCA-ACT-GCA-AAT-AGC-GGA-TCC-GCG-GAA-CAG-GTT-CAA-TCA-AGA-TCA-AGA-AGA-TCA-CTC-ACA-GAG-GGA-AGT-GAA-ACA-CCT-GCA-ACC-GTC-GAT-TTG-TTT-GCC-TTC-ACC-CTT-GAT-GGT-GGT-AAA-AGA-ATT-GAA-GTG-GCT-GTA-CCA-AAC-AAC-GAG-GAT-GCA-TCC-AAA-AGA-ACC-GAG-TAC-AGT-TTG-GTT-GCA-AAC-GAT-AAG-CCT-TTC-TAT-ACC-GGC-GCA-AAT-AGC-GGC-ACC-GAA-AAT-GGT-GTC-TAC-AAG-TTG-AAT-GAG-AAC-GGA-GAC-TTG-GTN-GAC-AAG-GAC-AAT-AAA-GTT-CTT-TTG-AGR-SGT-3′	MW480843	*C. parvum*	IIe
30	5′-TGW-TGA-GGG-ATC-ATC-ATC-ATC-ATC-ATC-ATC-ATC-ATC-ATC-ATC-ATC-ATC-ATC-ATC-ATC-ATC-ATC-AAC-ATC-GAC-CGT-CGC-ACC-AGC-TCC-AAA-GAA-AGA-AAG-AAC-TGT-AGA-GGG-CGG-CAC-GGA-AGG-AAA-GAA-CGA-AGA-AAG-CAG-TCC-AGG-TTC-TGA-AGA-ACA-AGA-CGG-TGG-TAA-GGA-GGA-CGG-TGG-TAA-GGA-AAA-CGG-TGA-AGG-AGA-CAC-AGT-AGA-CGG-GGA-ACA-AAC-CGG-GAG-TGG-TTC-TCA-AGT-TAC-TCC-ATC-TGG-AAG-TGC-CGG-CAC-AGC-TAC-CGA-GTC-CAC-AGC-AAC-TAC-TAC-TCC-AAA-GGA-AGA-ATG-TGG-TAC-TTC-ATT-TGT-CAT-GTG-GTT-CGA-GAA-AGG-CAC-CCC-GGT-TGC-GAC-CTT-GAA-GTG-TGG-TGA-TTA-CAC-TAT-CGT-CTA-TGC-ACC-TAT-AAA-AGA-TCA-AAC-AGA-TCC-CGC-ACC-AAG-ATA-TAT-CTC-TGG-TGA-AGT-TAC-ATC-TGT-ATC-CTT-TGA-AAA-GAG-TGA-AAG-TAC-AGT-TAC-AAT-CAA-GGT-TAA-TGG-TAA-AGA-GTT-CAG-CAC-TCT-CTC-TGC-TAA-CTC-AAG-TAG-TCC-AAC-TAA-AGA-TAA-CGG-TGA-ATC-TAG-TGA-CAG-TCA-GGT-TCA-ATC-AAG-ATC-AAG-AAG-ATC-ACT-CGC-AGA-GGA-GAA-TGG-TGA-AAC-AGT-TGC-AAC-AGT-TGA-TTT-GTT-TGC-CTT-TAC-TCT-TGA-TGG-TGG-TAG-AAG-AAT-TGA-AGT-GGC-TGT-GCC-AAA-GGA-CGA-AAA-TGC-AGA-CAA-AAG-AAG-CGA-GTA-CAG-TTT-GGT-TGC-AGA-CGA-TAA-GCC-TTT-CTA-TAC-CGG-CGC-AAA-CAG-TGG-CAT-CAC-CAA-TGG-TGT-CTA-CAA-ATT-GGA-TGA-GAA-TGG-AAA-CTT-GGK-3′	GU214345	*C. hominis*	Ia
31	5′-TTT-TTT-CYA-CCC-CAG-CCG-TTC-CAC-TCA-GAG-GCA-CTT-TAA-AGG-ATG-CTT-CTG-TTG-AGG-GCT-CAT-CAT-CAT-CAT-CAT-CAT-CAT-CAT-CAT-CAT-CGA-CCA-CCG-TCG-CAC-CAG-CTC-CAA-AGA-AAG-AAA-GAA-CTG-GAG-AGG-GCG-TAG-ATG-GAA-AGG-ACC-AAG-TAG-ATA-GTA-CAG-GTT-CTG-ATC-AGA-ACA-GTA-AAG-GAG-ACA-CTA-AAG-GAA-CCA-CAG-AAG-ATG-GTA-AAG-AGA-CCG-AAG-GTA-CTG-TTT-CCA-AAC-CCA-CTA-CTC-CAG-ATC-AAG-GTG-AGA-GCG-CAA-CTC-CCG-GAT-CCA-CGG-AAA-CTA-CTC-CAA-AGG-AAG-AAT-GCG-GTA-CTT-CAT-TTG-TAA-TGT-GGT-TCG-GAG-AAG-GTA-CCC-CAG-TTG-CGA-CCT-TGA-AGT-GTG-GTG-GTT-ACA-CTA-TCG-TCT-ATG-CAC-CTG-TAA-AGG-AAC-AAA-CAA-ATC-CCG-CAC-CAA-GAT-ATA-TCT-CTG-GTG-AGG-TAA-AAA-ATG-TAT-CCT-TCC-AAA-AAG-AAA-GTG-ATG-GTA-CAA-TTA-AAA-TCA-AGA-TTG-ACG-AAR-AGG-AGT-TCA-GTT-CTC-TCT-CTA-CTG-ACT-CAA-GCA-CTC-CAA-CTG-CAA-ATA-GCG-GAT-CCG-CGG-AAC-AGG-TTC-AAT-CAA-GAT-CAA-GAA-GAT-CAC-TCA-CAG-AGG-GAA-GTG-AAA-CAC-CTG-CAA-CCG-TCG-ATT-TGT-TTG-CCT-TCA-CCC-TTG-ATG-GTG-GTA-AAA-GAA-TTG-AAG-TGG-CTG-TAC-CAA-ACA-ACG-AGG-ATG-CAT-CCA-AAA-GAA-CCG-AGT-ACA-GTT-TGG-TTG-CAA-ACG-ATA-AGC-CTT-TCT-ATA-CCG-GCG-CAA-ATA-GCG-GCA-CCG-AAR-ATG-GTG-TCT-ACA-AGT-TGA-ATG-AGA-ACG-GAG-ACT-TGG-TTG-ACA-AGG-ACA-ATA-AAG-TTC-TTT-TGA-AGG-ATG-YGG-GG-3′	MW480843	*C. parvum*	IIe
32	5′-GAG-GAC-GAT-AGT-TCA-GAT-TCT-AGT-GGA-GGC-AGT-GTA-GGA-GGC-ACA-GAG-AGC-GGA-AGT-GCA-GGA-GGA-AAG-AAC-GAA-GAA-GAT-AGT-TCA-AGT-TCY-GGA-GGT-GCT-CAG-GAC-GGC-AGT-GGA-GGC-ACT-GCA-GAA-GGC-GCT-ACT-CAG-TCC-GAG-GST-ACT-GCT-TYT-CAA-GRT-GCT-CCA-TCT-CAA-GGT-TCT-GAC-AAA-ACT-ACC-GAG-TCC-ACA-CAA-ACT-ACT-CCA-AAG-GAA-GAG-TGC-GGT-ACT-TCG-TTT-GTA-ATG-TGG-TTC-GGT-GAA-GGT-ACC-CCG-GTY-GSG-ACC-TTG-AAG-TGT-GGT-GGT-TAC-ACT-ATC-GTY-TAT-GCA-CCT-GTA-AAG-GAT-CAA-GCA-AAT-CCC-GCA-CCA-AGA-TAT-ATC-TCT-GGT-GAA-GTA-AAG-AAT-GTA-TCC-TTC-CAA-AAA-GAA-AGT-GAT-AAT-ACA-ATT-AAA-ATC-AAG-GTT-GAC-GGT-CAG-GAT-TTC-AGC-ACT-CTC-TCT-GCT-AGC-TCA-AGT-AGT-CCA-ACC-GAA-AAT-AAA-GGT-GAG-TMT-GGC-AAT-CAG-GTT-GAG-TCA-AGA-TCA-AGA-AGA-TCA-CTC-ACA-GAG-GAA-ACT-AGT-GAA-ACT-GCA-ACC-GTC-GAT-TTG-TTT-GCC-TTT-ACC-CTT-AAT-GGT-GGT-AAG-AGA-ATT-GAA-GTG-GCT-GTG-CCA-AAC-GCC-GAA-GAA-ACA-TCG-AAA-AGA-GAC-AAG-TAC-AGT-TTG-GTT-GCA-GAC-GAT-AGT-GCT-TTC-3′	PV261913	*C. hominis*	Id
33	5′-TTT-TYA-CCC-CAG-CCG-TCC-CAC-TCA-GAG-GCA-CCT-TGA-AGG-ATG-TTT-CTG-TTG-AGG-GCT-CAT-CAT-CAT-CTT-CAT-CAT-CGT-CTT-CAT-CTT-CAT-CAT-CAT-CAT-CGT-CGT-CAA-CAA-CCC-CAG-CAC-CAG-CTT-CAA-AGA-AGG-TAA-GAG-AAG-CAG-AAG-GCA-GTG-AAG-AAA-AGG-ACA-GCG-AAG-AAA-AGG-ACA-GTG-AAG-AAA-AGG-GCA-GTG-AAG-AAG-GTA-GCC-AAA-CTC-CCG-CTA-GTC-CTG-GAG-GTG-GAG-GGG-TGA-GTG-AAG-GAG-ATA-CTC-AAG-GTG-ACT-CTA-AAG-GAG-ACG-GAG-TTA-GTT-CAG-ATG-AGA-ACC-AAA-GTC-AAG-GTG-GGG-ACG-CTA-CTC-CCG-GAT-CTA-GCA-CCC-AAA-CTC-AAG-CTA-CTG-AAA-AAG-AAC-CCG-GAT-CTT-CAG-AAG-CTA-CTC-CAA-AGG-AAG-AGT-GCG-GTA-CTT-CAT-TTG-TAA-TGT-GGT-TCG-GAC-AGG-GTG-TTC-CAG-TTG-TAA-CTT-TGA-AGT-GTG-GTG-GTT-ATA-CTA-TGG-TCT-ATG-CAC-CAG-AAA-ATG-GCA-AAA-CAG-ATC-CCG-CAC-CAA-GAT-ATA-TCT-CTG-GTA-AAG-TTT-CAA-CCG-TAG-ACT-TTG-AAA-AAC-AAG-ATA-GTA-CAG-TTA-AAA-TCA-AGG-TTA-ATG-GTG-TGG-AGT-TCA-GCA-CTC-TCT-CTA-CTA-GCT-CAA-GTA-ATC-CAA-CTG-AAA-ATA-GCG-GAT-CTG-AGA-GCC-AGG-CTC-AAT-CAA-GAT-CAA-GAA-GAT-CAC-TCG-CAG-AGG-ATG-GTA-CTG-AGA-CTG-CTG-CAA-CCG-TCG-ATT-TGA-TTG-CCT-TCA-CCC-TTC-AAG-GTG-GTA-AAA-GAA-TCG-AAG-TCG-CTG-TGC-CAA-GTG-ACG-AAG-ATG-TAT-CCA-AGA-GAA-ACA-AGT-ACA-GTT-TGG-TTG-CAG-GCG-ATA-AGA-CTT-TCT-ATA-CCG-GCG-CAA-ATA-GCG-GTA-ATA-CCG-ACG-GTA-TCT-ACA-GGT-TGA-ATG-ATG-ATG-GAG-ACT-TGG-TGG-ACA-AGA-ACA-ACA-ACG-TTC-TTT-TGA-AGA-BG-3′	ON863660	*C. hominis*	Ie
34	5′-CCC-CAG-CCG-TTC-CAC-TCA-GAG-GCA-CCT-TGA-AGG-ATG-TTT-CTG-TTG-AGG-GCT-CAT-CAT-CAT-CTT-CAT-CAT-CGT-CTT-CAT-CTT-CAT-CAT-CAT-CAT-CGT-CGT-CAA-CAA-CCC-CAG-CAC-CAG-CTT-CAA-AGA-AGG-TAA-GAG-AAG-CAG-AAG-GCA-GTG-AAG-AAA-AGG-ACA-GCG-AAG-AAA-AGG-ACA-GTG-AAG-AAA-AGG-GCA-GTG-AAG-AAG-GTA-GCC-AAA-CTC-CCG-CTA-GTC-CTG-GAG-GTG-GAG-GGG-TGA-GTG-AAG-GAG-ATA-CTC-AAG-GTG-ACT-CTA-AAG-GAG-ACG-GAG-TTA-GTT-CAG-ATG-AGA-ACC-AAA-GTC-AAG-GTG-GGG-ACG-CTA-CTC-CCG-GAT-CTA-GCA-CCC-AAA-CTC-AAG-CTA-CTG-AAA-AAG-AAC-CCG-GAT-CTT-CAG-AAG-CTA-CTC-CAA-AGG-AAG-AGT-GCG-GTA-CTT-CAT-TTG-TAA-TGT-GGT-TCG-GAC-AGG-GTG-TTC-CAG-TTG-TAA-CTT-TGA-AGT-GTG-GTG-GTT-ATA-CTA-TGG-TCT-ATG-CAC-CAG-AAA-ATG-GCA-AAA-CAG-ATC-CCG-CAC-CAA-GAT-ATA-TCT-CTG-GTA-AAG-TTT-CAA-CCG-TAG-ACT-TTG-AAA-AAC-AAG-ATA-GTA-CAG-TTA-AAA-TCA-AGG-TTA-ATG-GTG-TGG-AGT-TCA-GCA-CTC-TCT-CTA-CTA-GCT-CAA-GTA-ATC-CAA-CTG-AAA-ATA-GCG-GAT-CTG-AGA-GCC-AGG-CTC-AAT-CAA-GAT-CAA-GAA-GAT-CAC-TCG-CAG-AGG-ATG-GTA-CTG-AGA-CTG-CTG-CAA-CCG-TCG-ATT-TGA-TTG-CCT-TCA-CCC-TTC-AAG-GTG-GTA-AAA-GAA-TCG-AAG-TCG-CTG-TGC-CAA-GTG-ACG-AAG-ATG-TAT-CCA-AGA-GAA-ACA-AGT-ACAG-TTT-GGT-TGC-AGG-CGA-TAA-GAC-TTT-CTA-TAC-CGG-CGC-AAA-TAG-CGG-TAA-TAC-CGA-CGG-TAT-CTA-CAG-GTT-GAA-TGA-TGA-TGG-AGA-CTT-GGT-GGA-CAA-GAA-CAA-CAA-CGT-TCT-TTG-AAG-WSG-GGG-3′	ON863660	*C. hominis*	Ie
35	5′-CAC-AGC-CGT-TCY-KCT-CAG-AGG-CAC-CTT-GAA-GGA-TGT-TTC-TGT-TGA-GGR-CTC-ATC-ATC-ATC-ATC-ATC-ATC-ATC-ATC-ATC-ATC-ATC-ATC-ATC-ATC-AAC-GAC-CGT-CGC-ACC-AGC-TTC-AAA-TAA-GGC-AAG-AAC-TGG-AGA-GGA-CAC-AGG-ACG-AAG-CGA-AGG-AAG-TCA-AGG-TTC-TGA-AGA-ACA-CCA-AGA-CGG-AGA-GGA-CGA-TAG-TTC-AGA-TTC-TAG-TGG-AGG-CAG-TGT-AGG-AGG-CAC-AGA-GAG-CGG-AAG-TGC-AGG-AGG-AAA-GAA-CGA-AGA-AGA-TAG-TTC-AAG-TTC-TGG-AGG-TGC-TCA-GGA-CGG-CAG-TGG-AGG-CAC-TGC-AGA-AGG-CGC-TAC-TCA-GTC-CGA-GGC-TAC-TGC-TTC-TCA-AGG-TGC-TCC-ATC-TCA-AGG-TTC-TGA-CAA-AAC-TAC-CGA-GTC-CAC-ACA-AAC-TAC-TCC-AAA-GGA-AGA-GTG-CGG-TAC-TTC-GTT-TGT-AAT-GTG-GTT-CGG-TGA-AGG-TAC-CCC-GGT-TGC-GAC-CTT-GAA-GTG-TGG-TGG-TTA-CAC-TAT-CGT-CTA-TGC-ACC-TGT-AAA-GGA-TCA-AGC-AAA-TCC-CGC-ACC-AAG-ATA-TAT-CTC-TGG-TGA-AGT-AAA-GAA-TGT-ATC-CTT-CCA-AAA-AGA-AAG-TGA-TAA-TAC-AAT-TAA-AAT-CAA-GGT-TGA-CGG-TCA-GGA-TTT-CAG-CAC-TCT-CTC-TGC-TAG-CTC-AAG-TAG-TCC-AAC-CGA-AAA-TAA-AGG-TGA-GTC-TGG-CAA-TCA-GGT-TGA-GTC-AAG-ATC-AAG-AAG-ATC-ACT-CAC-AGA-GGA-AAC-TAG-TGA-AAC-TGC-AAC-CGT-CGA-TTT-GTT-TGC-CTT-TAC-CCT-TAA-TGG-TGG-TAA-GAG-AAT-TGA-AGT-GGC-TGT-GCC-AAA-CGC-CGA-AGA-AAC-ATC-GAA-AAG-AGA-CAA-GTA-CAG-TTT-GGT-TGC-AGA-CGA-TAG-TGC-TTT-CTA-TAC-CGG-CAA-AAA-TAG-CGG-CAG-CAC-CGA-TGG-TGT-CTA-CAA-GTT-GAA-TGA-GAA-CGG-AGA-CTT-AGT-GA-3′	MK982538	*C. hominis*	Id
36	5′-KKT-TTT-TCT-CAG-CCC-CAG-CCG-TTC-CAC-TCA-GAG-GCA-CTT-TAA-AGG-ATG-TTT-CTG-TTG-AGA-GCT-CAT-CGT-CAT-CAT-CGT-CAT-CGT-CAA-CAA-CCC-CCG-CAC-CAG-CTC-CAA-AGA-AGG-TAA-GAG-AAA-GCG-AAG-AAG-GGA-AGA-ACA-GTG-AAG-ATA-GTC-AAA-CTC-CCG-CTA-GTC-CTG-GAA-GTG-GTT-CTC-AGG-ATA-GCT-CTA-AAG-GAG-ACG-AAG-TTG-TAG-ATG-GAG-GCG-TTT-CCG-GAT-CTA-GTA-CCC-CAA-CTC-AAG-CTG-CTG-AAA-AGG-AGC-CCG-AAA-CTC-CAG-AAT-CTA-CTC-CAA-AGG-AAG-AAT-GTG-GTA-CTT-CAT-TTA-TAA-TGT-GGT-TCG-GAG-AAG-GTA-CTC-CAG-CCA-CAA-CTT-TGA-AGT-GCG-GTG-GCT-ACA-CTA-TCG-TCT-ATG-CAC-CAG-AAA-AGG-ATA-ATA-AAG-AAC-CCG-CAC-CAA-GAT-ACA-TCT-CTG-GTG-ATG-TTA-AGG-CTG-TAA-CCT-TTG-AAA-AGG-GAG-AAG-ATA-ATA-CAG-TTA-AAA-TCA-AGG-TTG-ATG-GTA-GGG-AGT-TCA-GTA-CTC-TCT-CTT-CTA-GCT-CAA-GCA-GTC-CAA-CTG-AAA-ATA-GCG-GAT-CTG-AGA-GCC-AGG-CTC-AAT-CAA-GAT-CAA-GAA-GAT-CAC-TCA-CAG-AGG-GAA-GTG-AAA-CAC-CTG-CAA-CCG-TCG-ATT-TGT-TTG-CCT-TCA-CCC-TTC-AAG-GTG-GTA-AAA-GAA-TCG-AAG-TCG-CTG-TGC-CAA-GTG-ACG-TAG-ATG-CAT-CCA-AGA-GAA-ACA-AGT-ACA-GTT-TGG-TTG-CAG-GCG-ATA-AGA-CTT-TCT-ATA-CCG-GCG-CAA-ATA-GCG-GTA-ATA-CCG-ACG-GTA-TCT-ACA-GGT-TGA-ATG-ATG-ATG-GAG-ACT-TGG-TGG-ACA-AGA-ACA-ACA-ACG-TTC-TTT-TGA-AGG-ATK-3′	JF802124	*C. parvum*	IIc
37	5′-TCA-GCC-CCA-GCC-GTT-CCA-CTC-AGA-GGC-ACC-TTG-AAA-GAT-GTT-TCT-GTT-GAG-AGC-TCA-TCA-TCA-TCA-TCA-TCA-TCA-TCG-TCA-TCA-TCA-TCG-TCA-TCA-TCG-TCA-ACA-ACA-ACC-CCC-GCA-CCA-GCT-CCA-AAG-AAG-GCA-AGA-GAA-GCA-GAT-GGC-GGA-GAA-GAA-AAG-AAC-AAT-GAA-GAA-AGC-CAA-ACT-CCC-GCT-AGT-CCT-GGA-AGT-GGT-GGG-GTG-AGT-GAA-GGA-CAA-GAT-ACT-CAA-GGT-GGC-TCC-AAA-GGA-GAC-GCT-GAG-GAA-GGC-ACT-GAA-GAC-AAT-GAA-CAA-GCC-GAT-GAG-AGT-GCT-ACC-CAA-CCT-TCT-ACC-CCA-GGT-CAA-GGC-TCC-GTT-AAA-ACC-GAA-TCC-ACA-GAA-ACT-ACT-CCA-AAG-GAG-AAG-TGC-GGT-ACT-TCA-TTT-GTT-ATG-TGG-TTC-GGA-CAG-GGT-GTT-CCA-GTC-GCA-ACT-TTG-AAG-TGC-GGT-GAC-TAT-ACT-ATG-GTC-TAT-GCA-CCA-GAA-AAG-GAC-AAA-ACA-GAT-CCC-GCA-CCA-AGA-TAT-ATC-TCT-GGT-GAA-GTT-ACA-ACC-GTA-ACC-TTT-GAT-AAA-CAA-GAG-AGT-ACA-GTT-ACA-ATC-AAG-GTT-AAT-AAT-GTA-GAG-TTC-GGC-ACT-CTC-TCT-ACT-AGC-TCA-AGT-AAA-CCA-ACT-GAA-AAT-AAA-GGT-GAG-TCT-AGC-GAT-CAG-GTT-GGG-TCA-AGA-TCA-AGA-AGA-TCA-CTC-ACA-GAG-GAA-ACT-AGT-GAA-ACT-GCA-ACC-GTC-GAT-TTG-TTT-GCC-TTT-ACC-CTT-GAT-GGT-GGT-AAA-AGA-ATT-GAA-GTG-GCT-GTA-CCA-AGT-GAC-GAA-GAT-GTA-TCC-AAG-AGA-AAC-AAG-TAC-AGT-TTG-GTT-GCA-AAC-GAT-AAG-ACT-TTC-TAT-ACC-GGC-GCA-AAT-AGC-GGT-AAT-ACC-GAC-GGT-ATC-TAC-AGG-TTG-AAT-GAT-AAT-GGA-GAC-TTG-GTG-GAC-AAG-AAC-AAC-AAC-GTT-CTT-TTG-AAG-GAT-G-3′	MT053132	*C. hominis*	Ib
38	5′-YTT-TTT-TTY-ACC-CAG-CCG-TTC-CAC-TCA-GAG-GCA-CTT-TAA-AGG-ATG-TTT-CTG-TTG-AGA-GCT-CAT-CGT-CAT-CAT-CGT-CAT-CGT-CAA-CAA-CCC-CCG-CAC-CAG-CTC-CAA-AGA-AGG-TAA-GAG-AAA-GCG-AAG-AAG-GGA-AGA-ACA-GTG-AAG-ATA-GTC-AAA-CTC-CCG-CTA-GTC-CTG-GAA-GTG-GTT-CTC-AGG-ATA-GCT-CTA-AAG-GAG-ACG-AAG-TTG-TAG-ATG-GAG-GCG-TTT-CCG-GAT-CTA-GTA-CCC-CAA-CTC-AAG-CTG-CTG-AAA-AGG-AGC-CCG-AAA-CTC-CAG-AAT-CTA-CTC-CAA-AGG-AAG-AAT-GTG-GTA-CTT-CAT-TTA-TAA-TGT-GGT-TCG-GAG-AAG-GTA-CTC-CAG-CCA-CAA-CTT-TGA-AGT-GCG-GTG-GCT-ACA-CTA-TCG-TCT-ATG-CAC-CAG-AAA-AGG-ATA-ATA-AAG-AAC-CCG-CAC-CAA-GAT-ACA-TCT-CTG-GTG-ATG-TTA-AGG-CTG-TAA-CCT-TTG-AAA-AGG-GAG-AAG-ATA-ATA-CAG-TTA-AAA-TCA-AGG-TTG-ATG-GTA-GGG-AGT-TCA-GTA-CTC-TCT-CTT-CTA-GCT-CAA-GCA-GTC-CAA-CTG-AAA-ATA-GCG-GAT-CTG-AGA-GCC-AGG-CTC-AAT-CAA-GAT-CAA-GAA-GAT-CAC-TCA-CAG-AGG-GAA-GTG-AAA-CAC-CTG-CAA-CCG-TCG-ATT-TGT-TTG-CCT-TCA-CCC-TTC-AAG-GTG-GTA-AAA-GAA-TCG-AAG-TCG-CTG-TGC-CAA-GTG-ACG-TAG-ATG-CAT-CCA-AGA-GAA-ACA-AGT-ACA-GTT-TGG-TTG-CAG-GCG-ATA-AGA-CTT-TCT-ATA-CCG-GCG-CAA-ATA-GCG-GTA-ATA-CCG-ACG-GTA-TCT-ACA-GGT-TGA-ATG-ATG-ATG-GAG-ACT-TGG-TGG-ACA-AGA-ACA-ACA-ACG-TTC-TTT-TGA-GAT-S-3′	JF802124	*C. parvum*	IIc
39	5′-TTT-TTY-ACC-CCA-GCC-GTT-CCM-YTC-AGA-GGC-ACC-TTG-AAA-GAT-GTT-TCT-GTT-GAG-AGC-TCA-TCA-TCA-TCA-TCA-TCA-TCA-TCG-TCA-TCA-TCA-TCG-TCA-TCA-TCG-TCA-ACA-ACA-ACC-CCC-GCA-CCA-GCT-CCA-AAG-AAG-GCA-AGA-GAA-GCA-GAT-GGC-GGA-GAA-GAA-AAG-AAC-AAT-GAA-GAA-AGC-CAA-ACT-CCC-GCT-AGT-CCT-GGA-AGT-GGT-GGG-GTG-AGT-GAA-GGA-CAA-GAT-ACT-CAA-GGT-GGC-TCC-AAA-GGA-GAC-GCT-GAG-GAA-GGC-ACT-GAA-GAC-AAT-GAA-CAA-GCC-GAT-GAG-AGT-GCT-ACC-CAA-CCT-TCT-ACC-CCA-GGT-CAA-GGC-TCC-GTT-AAA-ACC-GAA-TCC-ACA-GAA-ACT-ACT-CCA-AAG-GAG-AAG-TGC-GGT-ACT-TCA-TTT-GTT-ATG-TGG-TTC-GGA-CAG-GGT-GTT-CCA-GTC-GCA-ACT-TTG-AAG-TGC-GGT-GAC-TAT-ACT-ATG-GTC-TAT-GCA-CCA-GAA-AAG-GAC-AAA-ACA-GAT-CCC-GCA-CCA-AGA-TAT-ATC-TCT-GGT-GAA-GTT-ACA-ACC-GTA-ACC-TTT-GAT-AAA-CAA-GAG-AGT-ACA-GTT-ACA-ATC-AAG-GTT-AAT-AAT-GTA-GAG-TTC-GGC-ACT-CTC-TCT-ACT-AGC-TCA-AGT-AAA-CCA-ACT-GAA-AAT-AAA-GGT-GAG-TCT-AGC-GAT-CAG-GTT-GGG-TCA-AGA-TCA-AGA-AGA-TCA-CTC-ACA-GAG-GAA-ACT-AGT-GAA-ACT-GCA-ACC-GTC-GAT-TTG-TTT-GCC-TTT-ACC-CTT-GAT-GGT-GGT-AAA-AGA-ATT-GAA-GTG-GCT-GTA-CCA-AGT-GAC-GAA-GAT-GTA-TCC-AAG-AGA-AAC-AAG-TAC-AGT-TTG-GTT-GCA-AAC-GAT-AAG-ACT-TTC-TAT-ACC-GGC-GCA-AAT-AGC-GGT-AAT-ACC-GAC-GGT-ATC-TAC-AGG-TTG-AAT-GAT-AAT-GGA-GAC-TTG-GTG-GAC-AAG-AAC-AAC-AAC-GTT-CTT-TTG-AAG-WWB-3′	MK105902	*C. hominis*	Ib
40	5′-TTY-ACC-CAG-CCG-TCC-CAC-TCA-GAG-GCA-CCT-TGA-AGG-ATG-TTT-CTG-TTG-AGG-GCT-CAT-CAT-CAT-CTT-CAT-CAT-CGT-CTT-CAT-CTT-CAT-CAT-CAT-CAT-CGT-CGT-CAA-CAA-CCC-CAG-CAC-CAG-CTT-CAA-AGA-AGG-TAA-GAG-AAG-CAG-AAG-GCA-GTG-AAG-AAA-AGG-ACA-GCG-AAG-AAA-AGG-ACA-GTG-AAG-AAA-AGG-GCA-GTG-AAG-AAG-GTA-GCC-AAA-CTC-CCG-CTA-GTC-CTG-GAG-GTG-GAG-GGG-TGA-GTG-AAG-GAG-ATA-CTC-AAG-GTG-ACT-CTA-AAG-GAG-ACG-GAG-TTA-GTT-CAG-ATG-AGA-ACC-AAA-GTC-AAG-GTG-GGG-ACG-CTA-CTC-CCG-GAT-CTA-GCA-CCC-AAA-CTC-AAG-CTA-CTG-AAA-AAG-AAC-CCG-GAT-CTT-CAG-AAG-CTA-CTC-CAA-AGG-AAG-AGT-GCG-GTA-CTT-CAT-TTG-TAA-TGT-GGT-TCG-GAC-AGG-GTG-TTC-CAG-TTG-TAA-CTT-TGA-AGT-GTG-GTG-GTT-ATA-CTA-TGG-TCT-ATG-CAC-CAG-AAA-ATG-GCA-AAA-CAG-ATC-CCG-CAC-CAA-GAT-ATA-TCT-CTG-GTA-AAG-TTT-CAA-CCG-TAG-ACT-TTG-AAA-AAC-AAG-ATA-GTA-CAG-TTA-AAA-TCA-AGG-TTA-ATG-GTG-TGG-AGT-TCA-GCA-CTC-TCT-CTA-CTA-GCT-CAA-GTA-ATC-CAA-CTG-AAA-ATA-GCG-GAT-CTG-AGA-GCC-AGG-CTC-AAT-CAA-GAT-CAA-GAA-GAT-CAC-TCG-CAG-AGG-ATG-GTA-CTG-AGA-CTG-CTG-CAA-CCG-TCG-ATT-TGA-TTG-CCT-TCA-CCC-TTC-AAG-GTG-GTA-AAA-GAA-TCG-AAG-TCG-CTG-TGC-CAA-GTG-ACG-AAG-ATG-TAT-CCA-AGA-GAA-ACA-AGT-ACA-GTT-TGG-TTG-CAG-GCG-ATA-AGA-CTT-TCT-ATA-CCG-GCG-CAA-ATA-GCG-GTA-ATA-CCG-ACG-GTA-TCT-ACA-GGT-TGA-ATG-ATG-ATG-GAG-ACT-TGG-TGG-ACA-AGA-ACA-ACA-ACG-TTC-TTT-GAG-RGK-3′	ON863660	*C. hominis*	Ie
41	5′-AGG-AGC-CAG-CAT-CTT-TCA-AGA-GAA-CTT-TGT-TGT-CST-TGT-CAA-CCA-AGT-TTC-CAT-TMT-CAT-CCA-ATT-TGT-AGA-CAC-CAT-TGG-TGA-TGC-CAC-TGT-TTG-CGC-CGG-TAT-AGA-AAG-GCT-TAT-CGT-CTG-CAA-CCA-AAC-TGT-ACT-CGC-TTC-TTT-TGT-CTG-CAT-YTT-CGT-CCT-TTG-GCA-CAG-CCA-CTT-CAA-TTC-TTC-TAC-CAC-CAT-CAA-GAG-TAA-AGG-CAA-ACA-AAT-CAA-CTG-TTG-CAA-CTG-TTT-CAC-CAT-TCT-CCT-CTG-CGA-GTG-ATC-TTC-TTG-ATC-TTG-ATT-GAA-CCT-GAC-TGT-CAC-TAG-ATT-CAC-CGT-TAT-CTT-TAG-TTG-GAC-TAC-TTG-AGT-TAG-CAG-AGA-GAG-TGC-TGA-ACT-CTT-TAC-CAT-TAA-CCT-TGA-TTG-TAA-CTG-TAC-TTT-CAC-TCT-TTT-CAA-AGG-ATA-CAG-ATG-TAA-CTT-CAC-CAG-AGA-TAT-ATC-TTG-GTG-CGG-GAT-CTG-TTT-GAT-CTT-TTA-TAG-GTG-CAT-AGA-CGA-TAG-TGT-AAT-CAC-CAC-ACT-TCA-AGG-TCG-CAA-CCG-GGG-TGC-CTT-TCT-CGA-ACC-ACA-TGA-CAA-ATG-AAG-TAC-CAC-ATT-CTT-CCT-TTG-GAG-TAG-TAG-TTG-CTG-TGG-ACT-CGG-TAG-CTG-TGC-CGG-CAC-TTC-CAG-ATG-GAG-TAA-CTT-GAG-AAC-CAC-TCC-CGG-TTT-GTT-CCC-CGT-CTA-CTG-TGT-CTC-CTT-CAC-CGT-TTT-CCT-TAC-CAC-CGT-CTT-CCT-TAC-CAC-CGT-CTT-GTT-CTT-CAG-AAC-CTG-GAC-TGC-TTT-CTT-CGT-TCT-TTC-CTT-CCG-TGC-CGC-CCT-CTA-CAG-TTC-TTT-CTT-TCT-TTG-GAG-CTG-GTG-CGA-CGG-TCG-ATG-TTG-ATG-ATG-ATG-ATG-ATG-ATG-ATG-ATG-ATG-ATG-ATG-ATG-ATG-ATG-ATC-CCT-CAR-CAG-3′	KY990897	*C. hominis*	Ia
42	5′-CTC-AKC-CGT-TCC-ACT-CAG-AGG-CAC-CTT-GAT-AGA-TGT-TTC-TGT-TGA-GAG-CTC-ATC-ATC-ATC-ATC-ATC-ATC-ATC-GTC-ATC-ATC-ATC-GTC-ATC-ATC-GTC-AAC-AAC-AAC-CCC-CGC-ACC-AGC-TCC-AAA-GAA-GGC-AAG-AGA-AGC-AGA-TGG-CGG-AGA-AGA-AAA-GAA-CAA-TGA-AGA-AAG-CCA-AAC-TCC-CGC-TAG-TCC-TGG-AAG-TGG-TGG-GGT-GAG-TGA-AGG-ACA-AGA-TAC-TCA-AGG-TGG-CTC-CAA-AGG-AGA-CGC-TGA-GGA-AGG-CAC-TGA-AGA-CAA-TGA-ACA-AGC-CGA-TGA-GAG-TGC-TAC-CCA-ACC-TTC-TAC-CCC-AGG-TCA-AGG-CTC-CGT-TAA-AAC-CGA-ATC-CAC-AGA-AAC-TAC-TCC-AAA-GGA-GAA-GTG-CGG-TAC-TTC-ATT-TGT-TAT-GTG-GTT-CGG-ACA-GGG-TGT-TCC-AGT-CGC-AAC-TTT-GAA-GTG-CGG-TGA-CTA-TAC-TAT-GGT-CTA-TGC-ACC-AGA-AAA-GGA-CAA-AAC-AGA-TCC-CGC-ACC-AAG-ATA-TAT-CTC-TGG-TGA-AGT-TAC-AAC-CGT-AAC-CTT-TGA-TAA-ACA-AGA-GAG-TAC-AGT-TAC-AAT-CAA-GGT-TAA-TAA-TGT-AGA-GTT-CGG-CAC-TCT-CTC-TAC-TAG-CTC-AAG-TAA-ACC-AAC-TGA-AAA-TAA-AGG-TGA-GTC-TAG-CGA-TCA-GGT-TGG-GTC-AAG-ATC-AAG-AAG-ATC-ACT-CAC-AGA-GGA-AAC-TAG-TGA-AAC-TGC-AAC-CGT-CGA-TTT-GTT-TGC-CTT-TAC-CCT-TGA-TGG-TGG-TAA-AAG-AAT-TGA-AGT-GGC-TGT-ACC-AAG-TGA-CGA-AGA-TGT-ATC-CAA-GAG-AAA-CAA-GTA-CAG-TTT-GGT-TGC-AAA-CGA-TAA-GAC-TTT-CTA-TAC-CGG-CGC-AAA-TAG-CGG-TAA-TAC-CGA-CGG-TAT-CTA-CAG-GTT-GAA-TGA-TAA-TGG-AGA-CTT-GGT-GGA-CAA-GAA-CAA-CAA-CGT-TCT-TTT-GAA-GGA-TGC-TGT-CT-3′	MT053132	*C. hominis*	Ib
43	5′-CTT-CCG-CTG-TAW-TCT-CAG-CCC-CAG-CCG-TTC-CAC-TCA-GAG-GCA-CTT-TAA-AGG-ATG-TTT-CTG-TTG-AGA-GCT-CAT-CGT-CAT-CAT-CGT-CAT-CGT-CAA-CAA-CAA-CCC-CCG-CAC-CAG-CTC-CAA-AGA-AGG-TAA-GAG-AAA-GCG-AAG-AAG-GGA-AGA-ACA-GTG-AAG-ATA-GTC-AGA-CTC-CCG-CTA-GTC-CTG-GAA-GTG-ATT-CTC-AGG-ATA-GCT-CTA-AAG-GAG-ACG-AAG-CTG-TAG-ATG-GAT-CCG-CTT-CCG-GAT-CTA-GTA-CCC-CAA-CTC-AAG-CTG-CTG-AAA-AGG-AGC-CCG-AAA-CTC-CAG-AAT-CTA-CTC-CAA-AGG-AAG-AAT-GTG-GTA-CTT-CAT-TTA-TAA-TGT-GGT-TCG-GAG-AAG-GTA-CTC-CAG-CCA-CAA-CTT-TGA-AGT-GCG-GTG-GCT-ACA-CTA-TCG-TCT-ATG-CAC-CAG-AAA-AGG-ATA-ATA-AAG-AAC-CCG-CAC-CAA-GAT-ACA-TCT-CTG-GTG-ATG-TTA-AGG-CTG-TAA-CCT-TTG-AAA-AGG-GAG-AAG-ATA-ATA-CAG-TTA-AAA-TCA-AGG-TTG-ATG-GTA-AGG-AGT-TCA-GTA-CTC-TCT-CTT-CTA-GCT-CAA-GCA-GTC-CAA-CTG-AAA-ATA-ACG-GAT-CTA-CGG-GCC-AGG-TTG-CAT-CAA-GAT-CAA-GAA-GAT-CGC-TCT-CAG-AGG-AAA-ATA-GTG-AAA-CTG-CTG-CAA-CCG-TCG-ATT-TGT-TTG-CCT-TCA-CCC-TTG-ATG-GTG-GCC-GAA-GAA-TTG-AAG-TTG-CTG-TAC-CCA-GCG-TCG-AAG-ATG-CAA-CCA-AAA-GAG-ACA-AGT-ACA-GTT-TGG-TTG-CAA-ACG-GTA-AGC-CTT-TCT-ATA-CCG-GCG-CAA-ACA-GCG-GCA-CTA-CCA-ATG-GTG-TCT-ACA-GGT-TGA-ATG-AGA-ACG-GAG-ACT-TGG-TTG-ACA-AGG-ACA-ACA-CAG-TTC-TCT-TGA-AGG-ATG-CTG-TTT-YCY-TCT-G-3′	GU214366	*C. parvum*	IIc
44	5′-AGG-ACG-GTG-GTA-ATG-AAA-TCG-GTG-AAG-GAG-ACA-CAG-TAG-ACG-GCG-AAC-AAA-CCG-GGA-GTG-GTT-CTC-AAG-TTA-CTC-CAT-CTG-GAA-GTG-CCG-GCA-CAG-CTA-CCG-AGT-CCA-CAG-CAA-CTA-CTA-CTC-CAA-AGG-AAG-AAT-GTG-GTA-CTT-CAT-TTG-TCA-TGT-GGT-TCG-AGA-AAG-GCA-CCC-CGG-TTG-CGA-CCT-TGA-AGT-GTG-GTG-ATT-ACA-CTA-TCG-TCT-ATG-CAC-CTA-TAA-AAG-ATC-AAA-CAG-ATC-CCG-CAC-CAA-GAT-ATA-TCT-CTG-GTG-AAG-TTA-CAT-CTG-TAT-CCT-TTG-AAA-AGA-GTG-AAA-GTA-CAG-TTA-CAA-TCA-AGG-TTA-ATG-GTA-AAG-AGT-TCA-GCA-CTC-TCT-CTG-CTA-ACT-CAA-GTA-GTC-CAA-CTA-AAG-ATA-ACG-GTG-AAT-CTA-GTG-ACA-GTC-AGG-TTC-AAT-CAA-GAT-CAA-GAA-GAT-CAC-TCG-CAG-AGG-AGA-ATG-GTG-AAA-CAG-TTG-CAA-CAG-TTG-ATT-TGT-TTG-CCT-TTA-CTC-TTG-ATG-GTG-GTA-GAA-GAA-TTG-AAG-TGG-CTG-TGC-CAA-AGG-ACG-AAA-ATG-CAG-ACA-AAA-GAA-GCG-AGT-ACA-GTT-TGG-TTG-CAG-ACG-ATA-AGC-CTT-TCT-ATA-CCG-GCG-CAA-ACA-GTG-GCA-TCA-CCA-ATG-GTG-TCT-ACA-AAT-T-3′	OL598576	*C. hominis*	Ia
45	5′-GCT-TCC-GCT-GTA-TTC-TCA-GCC-CCA-GCC-GTT-CCA-CTC-AGA-GGC-ACC-TTG-AAA-GAT-GTT-TCT-GTT-GAG-AGC-TCA-TCA-TCA-TCA-TCA-TCA-TCA-TCG-TCA-TCA-TCA-TCG-TCA-TCA-TCG-TCA-ACA-ACA-ACC-CCC-GCA-CCA-GCT-CCA-AAG-AAG-GCA-AGA-GAA-GCA-GAT-GGC-GGA-GAA-GAA-AAG-AAC-AAT-GAA-GAA-AGC-CAA-ACT-CCC-GCT-AGT-CCT-GGA-AGT-GGT-GGG-GTG-AGT-GAA-GGA-CAA-GAT-ACT-CAA-GGT-GGC-TCC-AAA-GGA-GAC-GCT-GAG-GAA-GGC-ACT-GAA-GAC-AAT-GAA-CAA-GCC-GAT-GAG-AGT-GCT-ACC-CAA-CCT-TCT-ACC-CCA-GGT-CAA-GGC-TCC-GTT-AAA-ACC-GAA-TCC-ACA-GAA-ACT-ACT-CCA-AAG-GAG-AAG-TGC-GGT-ACT-TCA-TTT-GTT-ATG-TGG-TTC-GGA-CAG-GGT-GTT-CCA-GTC-GCA-ACT-TTG-AAG-TGC-GGT-GAC-TAT-ACT-ATG-GTC-TAT-GCA-CCA-GAA-AAG-GAC-AAA-ACA-GAT-CCC-GCA-CCA-AGA-TAT-ATC-TCT-GGT-GAA-GTT-ACA-ACC-GTA-ACC-TTT-GAT-AAA-CAA-GAG-AGT-ACA-GTT-ACA-ATC-AAG-GTT-AAT-AAT-GTA-GAG-TTC-GGC-ACT-CTC-TCT-ACT-AGC-TCA-AGT-AAA-CCA-ACT-GAA-AAT-AAA-GGT-GAG-TCT-AGC-GAT-CAG-GTT-GGG-TCA-AGA-TCA-AGA-AGA-TCA-CTC-ACA-GAG-GAA-ACT-AGT-GAA-ACT-GCA-ACC-GTC-GAT-TTG-TTT-GCC-TTT-ACC-CTT-GAT-GGT-GGT-AAA-AGA-ATT-GAA-GTG-GCT-GTA-CCA-AGT-GAC-GAA-GAT-GTA-TCC-AAG-AGA-AAC-AAG-TAC-AGT-TTG-GTT-GCA-AAC-GAT-AAG-ACT-TTC-TAT-ACC-GGC-GCA-AAT-AGC-GGT-AAT-ACC-GAC-GGT-ATC-TAC-AGG-TTG-AAT-GAT-AAT-GGA-GAC-TTG-GTG-GAC-AAG-AAC-AAC-AAC-GTT-CTT-TTG-AAG-GAT-GYT-GYT-TYC-YTC-TGC-AAT-AGG-ATT-CAG-ATA-CAT-CGG-TTC-CTT-CCA-G-3′	GU214349	*C. hominis*	Ib
46	5′-CGC-TGT-ATT-CTC-AGC-CCC-AGC-CGT-CCC-ACT-CAG-AGG-CAC-CTT-GAA-GGA-TGT-TTC-TGT-TGA-GGG-CTC-ATC-ATC-ATC-TTC-ATC-ATC-GTC-TTC-ATC-TTC-ATC-ATC-ATC-ATC-GTC-GTC-AAC-AAC-CCC-AGC-ACC-AGC-TTC-AAA-GAA-GGT-AAG-AGA-AGC-AGA-AGG-CAG-TGA-AGA-AAA-GGA-CAG-CGA-AGA-AAA-GGA-CAG-TGA-AGA-AAA-GGG-CAG-TGA-AGA-AGG-TAG-CCA-AAC-TCC-CGC-TAG-TCC-TGG-AGG-TGG-AGG-GGT-GAG-TGA-AGG-AGA-TAC-TCA-AGG-TGA-CTC-TAA-AGG-AGA-CGG-AGT-TAG-TTC-AGA-TGA-GAA-CCA-AAG-TCA-AGG-TGG-GGA-CGC-TAC-TCC-CGG-ATC-TAG-CAC-CCA-AAC-TCA-AGC-TAC-TGA-AAA-AGA-ACC-CGG-ATC-TTC-AGA-AGC-TAC-TCC-AAA-GGA-AGA-GTG-CGG-TAC-TTC-ATT-TGT-AAT-GTG-GTT-CGG-ACA-GGG-TGT-TCC-AGT-TGT-AAC-TTT-GAA-GTG-TGG-TGG-TTA-TAC-TAT-GGT-CTA-TGC-ACC-AGA-AAA-TGG-CAA-AAC-AGA-TCC-CGC-ACC-AAG-ATA-TAT-CTC-TGG-TAA-AGT-TTC-AAC-CGT-AGA-CTT-TGA-AAA-ACA-AGA-TAG-TAC-AGT-TAA-AAT-CAA-GGT-TAA-TGG-TGT-GGA-GTT-CAG-CAC-TCT-CTC-TAC-TAG-CTC-AAG-TAA-TCC-AAC-TGA-AAA-TAG-CGG-ATC-TGA-GAG-CCA-GGC-TCA-ATC-AAG-ATC-AAG-AAG-ATC-ACT-CGC-AGA-GGA-TGG-TAC-TGA-GAC-TGC-TGC-AAC-CGT-CGA-TTT-GAT-TGC-CTT-CAC-CCT-TCA-AGG-TGG-TAA-AAG-AAT-CGA-AGT-CGC-TGT-GCC-AAG-TGA-CGA-AGA-TGT-ATC-CAA-GAG-AAA-CAA-GTA-CAG-TTT-GGT-TGC-AGG-CGA-TAA-GAC-TTT-CTA-TAC-CGG-CGC-AAA-TAG-CGG-TAA-TAC-CGA-CGG-TAT-CTA-CAG-GTT-GAA-TGA-TGA-TGG-AGA-CTT-GGT-GGA-CAA-GAA-CAA-CAA-CGT-TCT-TTT-GAA-GGA-TGC-TGG-TTY-CCT-CTG-CAA-3′	GU214354	*C. hominis*	Ie
47	5′-TTT-TTT-CTC-CGC-TGT-ATT-CTC-AGC-CAC-AGC-CGT-TCC-ACT-CAG-AGG-CAC-TTT-AAA-GGA-TGT-TTC-TGT-TGA-GAG-CTC-ATC-GTC-ATC-ATC-GTC-ATC-GTC-AAC-AAC-AAC-CCC-CGC-ACC-AGC-TCC-AAA-GAA-GGT-AAG-AGA-AAG-CGA-AGA-AGG-GAA-GAA-CAG-TGA-AGA-TAG-TCA-GAC-TCC-CGC-TAG-TCC-TGG-AAG-TGA-TTC-TCA-GGA-TAG-CTC-TAA-AGG-AGA-CGA-AGC-TGT-AGA-TGG-ATC-CGC-TTC-CGG-ATC-TAG-TAC-CCC-AAC-TCA-AGC-TGC-TGA-AAA-GGA-GCC-CGA-AAC-TCC-AGA-ATC-TAC-TCC-AAA-GGA-AGA-ATG-TGG-TAC-TTC-ATT-TAT-AAT-GTG-GTT-CGG-AGA-AGG-TAC-TCC-AGC-CAC-AAC-TTT-GAA-GTG-CGG-TGG-CTA-CAC-TAT-CGT-CTA-TGC-ACC-AGA-AAA-GGA-TAA-TAA-AGA-ACC-CGC-ACC-AAG-ATA-CAT-CTC-TGG-TGA-TGT-TAA-GGC-TGT-AAC-CTT-TGA-AAA-GGG-AGA-AGA-TAA-TAC-AGT-TAA-AAT-CAA-GGT-TGA-TGG-TAA-GGA-GTT-CAG-TAC-TCT-CTC-TTC-TAG-CTC-AAG-CAG-TCC-AAC-TGA-AAA-TAA-CGG-ATC-TAC-GGG-CCA-GGT-TGC-ATC-AAG-ATC-AAG-AAG-ATC-GCT-CTC-AGA-GGA-AAA-TAG-TGA-AAC-TGC-TGC-AAC-CGT-CGA-TTT-GTT-TGC-CTT-CAC-CCT-TGA-TGG-TGG-CCG-AAG-AAT-TGA-AGT-TGC-TGT-ACC-CAG-CGT-CGA-AGA-TGC-AAC-CAA-AAG-AGA-CAA-GTA-CAG-TTT-GGT-TGC-AAA-CGG-TAA-GCC-TTT-CTA-TAC-CGG-CGC-AAA-CAG-CGG-CAC-TAC-CAA-TGG-TGT-CTA-CAG-GTT-GAA-TGA-GAA-CGG-AGA-CTT-GGT-TGA-CAA-GGA-CAA-CAC-AGT-TCT-CTT-GAA-GGA-TGC-TGG-TTT-CCT-CTG-CAT-TTG-GAC-TCA-GAT-ACA-TCG-TT-3′	GU214366	*C. parvum*	IIc
